# Regulating Type H Vessel Formation and Bone Metabolism via Bone‐Targeting Oral Micro/Nano‐Hydrogel Microspheres to Prevent Bone Loss

**DOI:** 10.1002/advs.202207381

**Published:** 2023-03-26

**Authors:** Junjie Li, Gang Wei, Gongwen Liu, Yawei Du, Ruizhi Zhang, Aifei Wang, Baoshan Liu, Wenguo Cui, Peng Jia, Youjia Xu

**Affiliations:** ^1^ Department of Orthopaedics Second Affiliated Hospital of Soochow University Osteoporosis Research Institute of Soochow University No.1055 Sanxiang Road Suzhou 215000 P. R. China; ^2^ Department of Orthopaedics Shanghai Key Laboratory for Prevention and Treatment of Bone and Joint Diseases Shanghai Institute of Traumatology and Orthopaedics Ruijin Hospital Shanghai Jiao Tong University School of Medicine 197 Ruijin 2nd Road Shanghai 200025 P. R. China; ^3^ Department of Orthopaedics Land Force No.72 Group Army Hospital of PLA, No.9 Chezhan Road Huzhou 313000 P. R. China; ^4^ Department of Orthopaedics Suzhou TCM Hospital Affiliated to Nanjing University of Chinese Medicine No.18 Yangsu Road Suzhou 215000 P. R. China

**Keywords:** hydrogel microspheres, microfluidic, osteoporosis, polyhedral oligomeric silsesquioxane (POSS), vascularization

## Abstract

Postmenopausal osteoporosis is one of the most prevalent skeletal disorders in women and is featured by the imbalance between intraosseous vascularization and bone metabolism. In this study, a pH‐responsive shell–core structured micro/nano‐hydrogel microspheres loaded with polyhedral oligomeric silsesquioxane (POSS) using gas microfluidics and ionic cross‐linking technology are developed. This micro/nano‐hydrogel microsphere system (PDAP@Alg/Cs) can achieve oral delivery, intragastric protection, intestinal slow/controlled release, active targeting to bone tissue, and thus negatively affecting intraosseous angiogenesis and osteoclastogenesis. According to biodistribution data, PDAP@Alg/Cs can successfully enhance drug intestinal absorption and bioavailability through intestine adhesion and bone targeting after oral administration. In vitro and in vivo experiments reveal that PDAP@Alg/Cs promoted type H vessel formation and inhibited bone resorption, effectively mitigating bone loss by activating HIF‐1*α*/VEGF signaling pathway and promoting heme oxygenase‐1 (HO‐1) expression. In conclusion, this novel oral micro/nano‐hydrogel microsphere system can simultaneously accelerate intraosseous vascularization and decrease bone resorption, offering a brand‐new approach to prevent postmenopausal osteoporosis.

## Graphical abstract

In this study, the author construct a pH‐responsive shell‐core structured micro/nano‐hydrogel microspheres (PDAP@Alg/Cs) using gas microfluidics and ionic cross‐linking technique. The PDAP@Alg/Cs can achieve oral delivery, intragastric protection, intestinal slow/controlled release, and active targeting to bone tissue. In conclusion, this novel oral PDAP@Alg/Cs can simultaneously accelerate intraosseous vascularization and decrease bone resorption, offering a brand‐new approach to prevent postmenopausal osteoporosis.

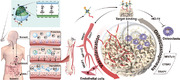

## 1 Introduction

Postmenopausal osteoporosis (PMOP) is typically accompanied by increased osteoclast activation and decreased blood circulation in the bone tissue, which displays a strong relationship with estrogen insufficiency.^[^ ^1^ ^]^ Recent research demonstrates that the prevention and treatment of PMOP significantly depend on the interaction between intraosseous angiogenesis, particularly type H vessel, and bone metabolism.^[^ ^2^ ^]^ However, traditional antiosteoporosis medicines, such as bisphosphonates, primarily inhibit osteoclast differentiation and function by reducing the release of platelet‐derived growth factor (PDGF‐BB) from osteoclast precursor cells, thereby inhibiting angiogenesis. Unfortunately, their long‐term use would induce harmful side effects including jaw osteonecrosis and atypical femur fractures, highlighting the increasing demands for safer antiosteoporosis medicines.^[^ ^3^ ^]^ Deferoxamine (DFO), the first Food and Drug Administration (FDA)‐approved Fe^3+^ chelator, is administered intravenously or intramuscularly to treat iron overload diseases caused by several reasons. Published research demonstrated that DFO stimulated vascular endothelial growth factor (VEGF) and hypoxia‐inducible factor‐1*α* (HIF‐1*α*) expression and encouraged angiogenesis in bone, including type H vessel.^[^ ^2^, ^4^ ^]^ Therefore, DFO has promising applications for treating PMOP. However, DFO is poorly absorbed orally with a short half‐life in vivo. Increasing the dosage or frequency of administration could result in several side effects, such as local swelling and pain from the injection, rash, growth retardation, bone disease, and auditory neurotoxicity.^[^ ^5^ ^]^ These issues significantly restrict the clinical translation of DFO. To our knowledge, only an ex vivo study to date reported that polymer micelles increased DFO permeability in the intestine of rats compared to free DFO, with a threefold increase in the optimal formulation.^[^ ^6^ ^]^ Usually, biomaterials are required as carriers for oral and bone‐targeting delivery, to prolong the half‐life and diminish the adverse effects associated with drug dose dependence.

For most chronic clinical conditions, oral administration is convenient, painless, and cost‐effective, and therefore has higher patient compliance. The complex physiological environment in the gastrointestinal tract severely restricts the effectiveness of oral drug delivery, including the acidic environment in the stomach, the abundance of digestive enzymes, and the mucus/mucosal barrier of the intestine, resulting in drug degradation, a short intestinal retention time, and low bioavailability.^[^ ^7^ ^]^ The efficacy of many oral administration methods has been tested, including nanoparticle carriers, ionic liquids, hydrogel microspheres, microneedle delivery systems, and microinjectors,^[^ ^8^ ^]^ though most of them were still limited to overcoming a single oral barrier. The results of the phase III clinical trial of oral salmon calcitonin prepared using Peptelligence technology to treat PMOP demonstrated that the oral delivery of salmon calcitonin was more effective in boosting bone mineral density (BMD) in the lumbar and hip compared to nasal spray delivery, though its bioavailability was only 1%.^[^ ^9^ ^]^

Apart from addressing the complex gastrointestinal environment, it must be considered how medicines can effectively target particular organs. Previous research found that an oligopeptide (Asp8) consisting of eight repetitive aspartic acid sequences specifically bound highly crystallized hydroxyapatite, thereby targeting the osteoclast‐covered bone resorption surface.^[^ ^10^ ^]^ Polyhedral oligomeric silsesquioxane (POSS) is a novel nanomaterial and has a unique Si‐O‐Si 3D cage‐shaped structure with eight arms that can be modified with organic groups of different functions or structures, enabling the original molecule to obtain higher thermal stability and stronger disaggregation effects. This makes POSS an ideal platform for creating intelligent single‐molecule organic–inorganic nanocomposite sensing materials.^[^ ^11^ ^]^ Additionally, POSS‐based organic–inorganic hybrid nanoparticles are widely utilized for drug delivery, tissue regeneration, and cell imaging, on account of their benefits of superior biocompatibility and biodegradability.^[^ ^12^ ^]^ Therefore, we synthesized organic‐inorganic hybrid nanoparticles by grafting DFO, Asp8, and polyethylene glycol 400 (PEG 400) to the POSS nanoplatform using click chemistry. To prevent PDAP NPs from degrading in the gastrointestinal tract and increase intestinal absorption, PDAP NPs were enclosed in pH‐responsive polymeric hydrogel microspheres (PDAP@Alg/Cs) made of alginate (Alg) and chitosan (Cs).

**Figure 1:**
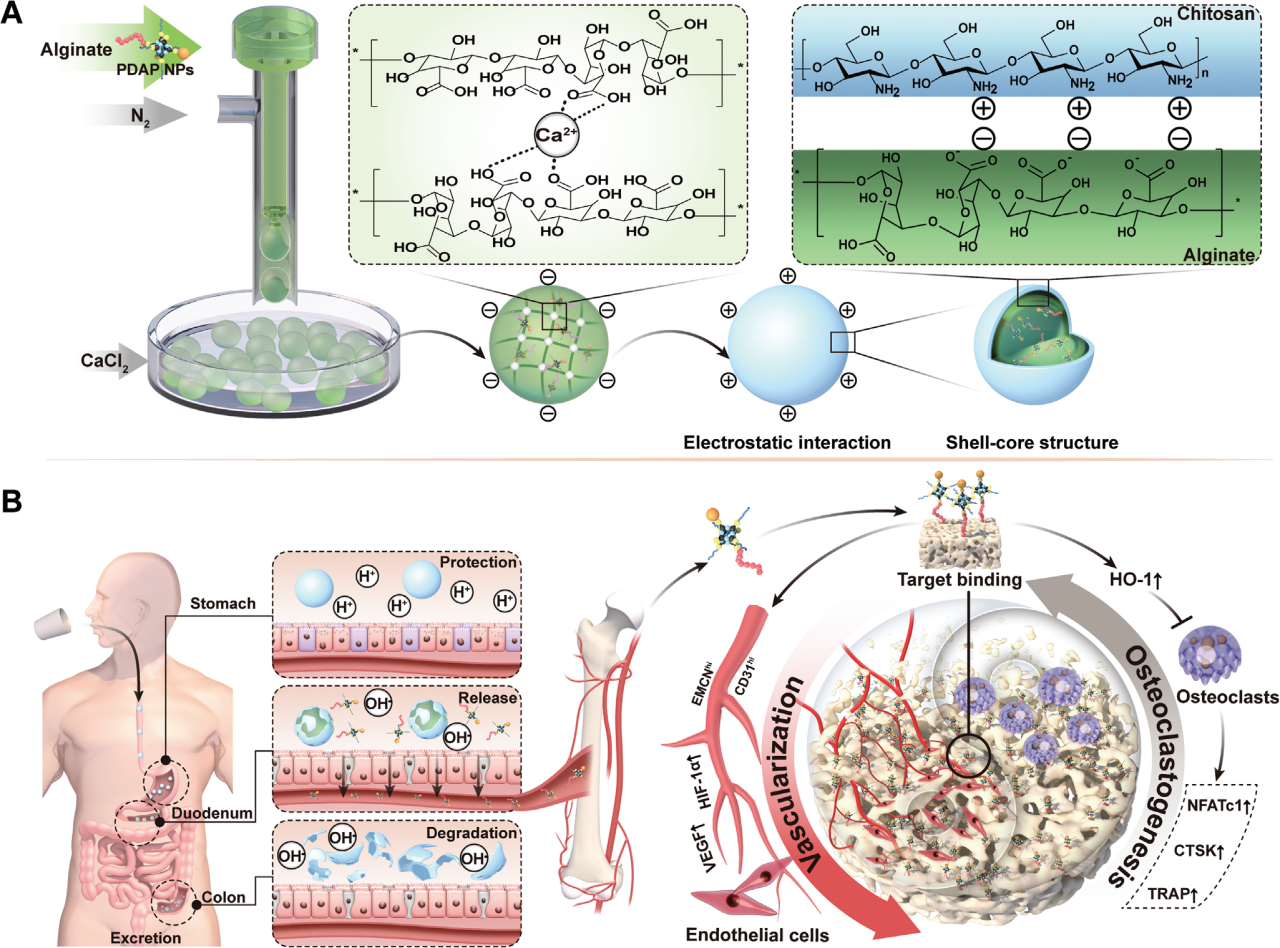
Schematic illustration of bone‐targeting oral micro/nano‐hydrogel microspheres for preventing bone loss. A) A pH‐responsive micro/nano‐hydrogel microsphere system encapsulating PDAP NPs (PDAP@Alg/Cs) was developed via gas microfluidic and ionic cross‐linking techniques. Calcium ion cross‐linked calcium alginate microspheres were located inside the microspheres and chitosan was bound to the surface of calcium alginate microspheres through electrostatic interaction to form complete shell–core structured hydrogel microspheres. B) After oral administration of PDAP@Alg/Cs microspheres, intragastric protection, intestinal slow/controlled release of PDAP NPs, and active targeting of bone tissue was achieved. PDAP NPs effectively mitigate bone loss by negatively affecting intraosseous angiogenesis and bone resorption.

Here, a pH‐responsive micro/nano‐hydrogel microsphere system encapsulating PDAP NPs was successfully developed via gas microfluidics and ionic cross‐linking techniques (**Figure** **1**). This PDAP@Alg/Cscan achieve oral delivery, intragastric protection, intestinal slow/controlled release, and active targeting to bone tissue. First, multifunctional organic–inorganic hybrid nanoparticles (PDAP NPs) were prepared via a Michael addition reaction; next, the core of hydrogel microspheres encapsulating PDAP NPs, calcium ion cross‐linked calcium alginate microspheres, were prepared via gas microfluidics and ion cross‐linking techniques. Calcium is used to prevent and treat osteoporosis. Finally, chitosan was bound to calcium alginate microspheres through electrostatic interaction on the surface, constituting complete shell–core structured hydrogel microspheres. In vitro tests revealed that PDAP@Alg/Cs released up to 80% in simulated intestine fluid (SIF), but only a tiny amount in simulated gastric fluid (SGF). Imaging results in mice demonstrated that the micro/nano‐hydrogel microsphere system exhibited intestinal adhesion and bone‐targeting delivery properties. By activating HIF‐1*α*/VEGF signaling pathway and inhibiting osteoclast‐related gene expression via promoting HO‐1 expression, PDAP NPs negatively affected intraosseous angiogenesis and bone resorption, significantly ameliorating bone loss, according to in vitro and in vivo tests. In summary, our study offers a brand‐new concept for the prevention and treatment of PMOP.

## 2 Results

### 2.1 Characterization of PDAP NPs

**Figure 2:**
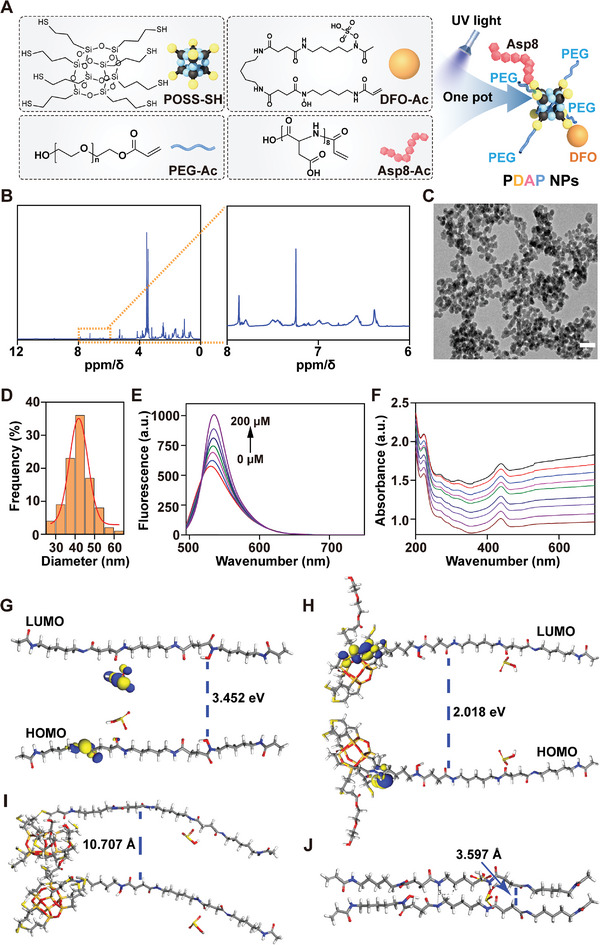
Characteristics of PDAP NPs. A) Schematic diagram of the synthesis of PDAP NPs. B) ^1^H NMR spectra of PDAP NPs. C) TEM image of PDAP NPs. Scale bars, 200 nm. D) Particle size distribution of PDAP NPs. E) Fluorescence spectra and F) UV–vis absorption spectrogram of PDAP NPs at different concentrations (≈0–200 µm). HOMO–LUMO orbital of G) POSS and H) POSS‐DFO. Molecular spacing of I) POSS‐DFO and J) POSS.

Sulfydryl‐modified POSS (POSS‐SH) was prepared by the hydrolytic condensation of 3‐mercaptopropyltriethoxysilane. The morphology of POSS‐SH was colorless transparent viscous liquid and its structure was confirmed to be pure using ^1^H nuclear magnetic resonance (^1^H‐NMR) and Fourier transform infrared (FTIR) spectroscopy (Figure S1A,S1B, Supporting Information). Then, DFO, Asp8, and PEG 400 were grafted to POSS via an environmentally‐friendly click chemistry reaction, and organic–inorganic nanoparticles (PDAP NPs) with bone‐targeting were synthesized using a one‐pot method (**Figure** **2**A). The structure was characterized by ^1^H NMR, FTIR, and MALDI‐TOF mass spectrometry. As seen in Figure 2B, the unreacted sulfhydryl peak was at 1.5 ppm and the characteristic peak of the benzene ring was between 6 and 8 ppm. According to MALDI‐TOF mass spectrometry, the molecular weight of PDAP NPs was ≈2485.2 (Figure S2, Supporting Information). The FTIR in Figure S3 (Supporting Information) further demonstrated the successful synthesis of PDAP NPs, of which the structural stoichiometric ratio could be precisely controlled base on the reactant proportion. Transmission electron microscopy (TEM) image showed that the prepared PDAP NPs had a homogeneous spherical morphology with an average particle size of 42 nm (Figure 2C,D). To better characterize the effect of the material in vivo, the fluorescence intensity was quantitatively tested using fluorescence spectroscopy under 365 nm UV light irradiation. The fluorescence intensity increased linearly with a concentration ranging from 0 to 200 µM (Figure 2E). Similarly, linear enhancement of the UV intensity from Figure 2F was consistent with fluorescence intensity. The changing trend of ultraviolet–visible (UV–Vis) and fluorescence spectrum is highly consistent, which not only indicates that the fluorescent group was successfully grafted on the PDAP NPs, but also the PDAP NPs have excellent nonquenching optical properties, providing a good basis for the tracing of the nanoparticles in vivo. Based on a density functional theory (DFT) calculation, we discovered that the highest occupied molecular orbital (HOMO) and lowest unoccupied molecular orbital (LUMO) orbital energy polar difference decreased from 3.452 (Figure 2G) to 2.018 eV (Figure 2H) and the average distance between molecules in the optimized geometries increased by 7.11 Å, as the DFO was modified with POSS (Figure 2I,J). These results suggested that POSS modification could form more stable compounds.

### 2.2 Characterization of PDAP@Alg/Cs

**Figure 3:**
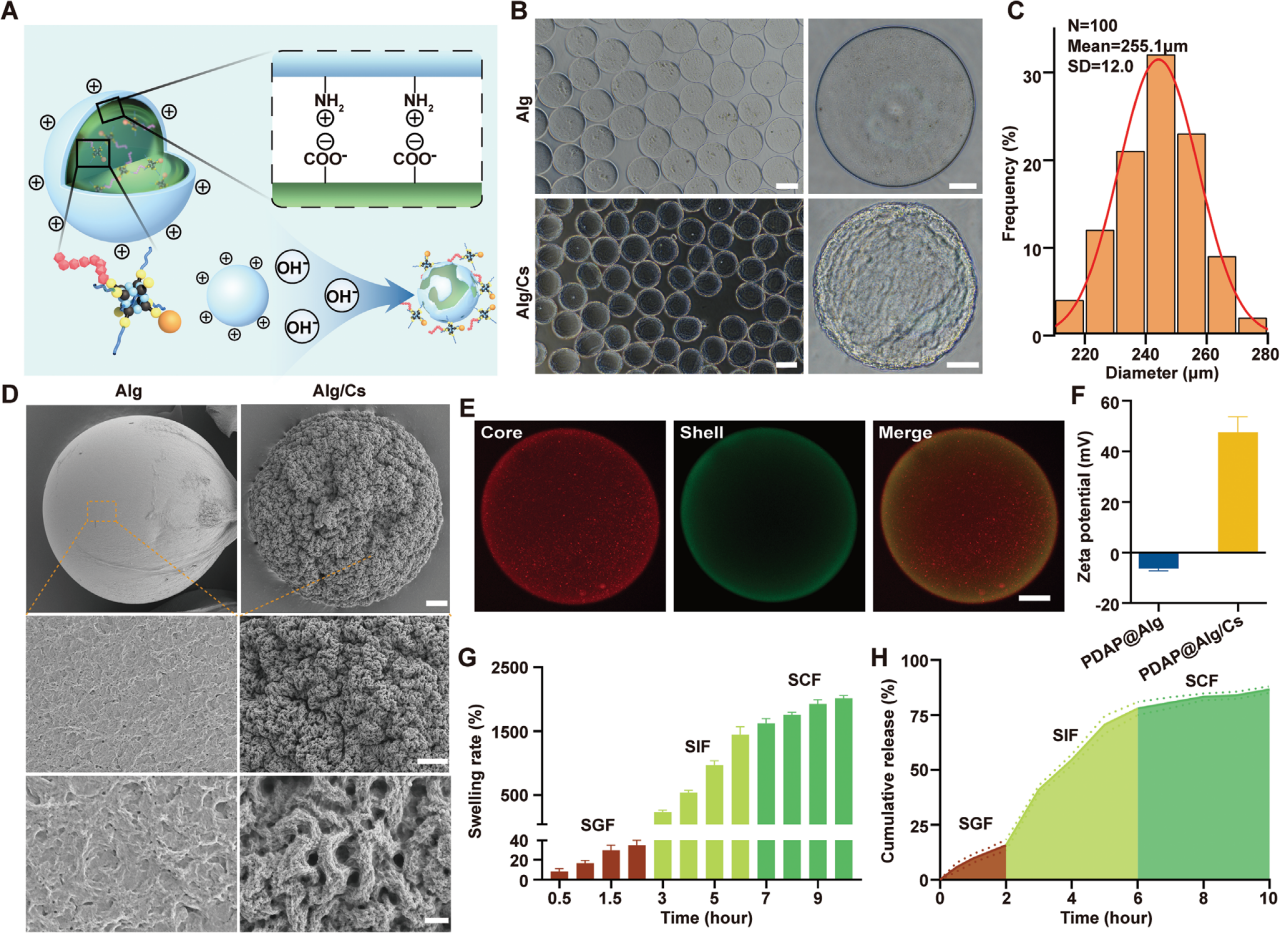
Characteristics of PDAP@Alg/Cs microspheres. A) Schematic diagram of the structure of PDAP@Alg/Cs microspheres. B) Optical microscopy images of PDAP@Alg and PDAP@Alg/Cs microspheres at different magnifications. Scale bars, 200 and 50 µm for left and right images, respectively. C) Size distribution of PDAP@Alg/Cs microspheres. D) SEM images of PDAP@Alg and PDAP@Alg/Cs microspheres at different magnifications. Scale bars, 10, 5, and 1 µm for top, middle, and bottom images, respectively. E) CLSM images of the shell‐core structure of Alg/Cs microspheres. Rho B(red) modified alginate (core), and FITC (green) modified chitosan (shell). Scale bars, 50 µm. F) Zeta potential of PDAP@Alg and PDAP@Alg/Cs (*n* = 3). G) Swelling rate of PDAP@Alg/Cs microspheres in SGF, SIF, and SCF (*n* = 3). H) Release curve of PDAP NPs from PDAP@Alg/Cs microspheres in SGF, SIF, and SCF.

In this study, sodium alginate hydrogel microspheres were prepared via a gas microfluidics technique. They were rapidly gelated in a calcium chloride solution, and the surface of calcium alginate microspheres was covered with chitosan based on electrostatic interaction (**Figure** **3**A; and Figure S4, Supporting Information). With a pump flow reaching 20 mL h^−1^, the prepared PDAP@Alg and PDAP@Alg/Cs hydrogel microspheres could be observed under optical microscopy and had excellent morphology and uniform particle size. Additionally, the yield was about 200 mg min^−1^. The average particle size of the PDAP@Alg/Cs was 255.1 ± 12.0 µm (Figure 3B,C). Scanning electron microscopy (SEM) was used to further observe the surface morphology of PDAP@Alg and PDAP@Alg/Cs hydrogel microspheres. PDAP@Alg/Cs hydrogel microspheres had a comparatively rough surface, which was different from PDAP@Alg hydrogel microspheres with a smooth surface (Figure 3D). Alginate and chitosan were labeled with rhodamine B (Rho B) and fluorescein isothiocyanate (FITC), respectively, and confocal laser scanning microscopy (CLSM) allowed us to observe distinct hydrogel microspheres with core–shell structure (Figure 3E; and Figure S5, Supporting Information). Additionally, PDAP@Alg were negatively charged and PDAP@Alg/Cs were positively charged, according to a zeta potential analysis (Figure 3F). Based on these data, chitosan was successfully wrapped on the surface of PDAP@Alg microspheres to create hydrogel microspheres with shell–core structure.

The retention time of drugs in the gastrointestinal tract depends on the composition of the substance, for example, high‐fat and high‐fiber foods, as well as high‐density objects having longer retention time. In general, the transit time through the stomach is typically 1–2 h, through the small intestine is 1–6 h, and through the colon is 1–3 days.^[^ ^13^ ^]^ We simulated the retention time of drugs in the gastrointestinal tract after they were orally administered. PDAP@Alg/Cs hydrogel microspheres slowly swelled in SGF, but their swelling ratios were significantly higher in SIF and simulated colon fluid (SCF) (Figure 3G). Only 16% of the PDAP NPs were released in the SGF after 2 h since PDAP@Alg/Cs hydrogel microspheres have a very low swelling rate in acidic environments, and were gradually released in SIF, reaching 80% after 4 h (Figure 3H). Even extended to 24 h, only 31% of PDAP NPs was released in SGF (Figure S6, Supporting Information). The PDAP@Alg/Cs hydrogel microspheres maintained a largely intact structure in SGF, deteriorated gradually in SIF, and virtually disappeared in SCF after 24 h (Figure S7, Supporting Information).

### 2.3 Biocompatibility and Targeting Properties

**Figure 4:**
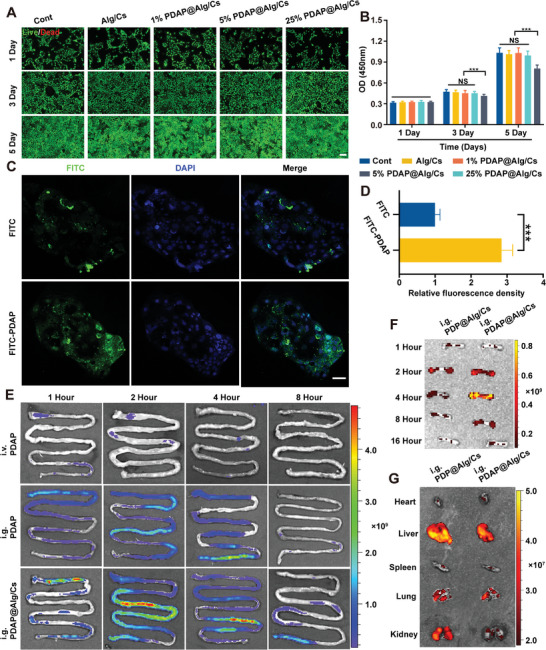
Biocompatibility and targeting properties. A) Live/Dead staining assay of Caco‐2 cells cocultured with different concentrations of PDAP@Alg/Cs extracts for 1, 3, and 5 days. Live cells were green and dead cells were red. Scale bars, 400 µm. B) CCK‐8 assay was used to detect the toxicity of different concentrations of PDAP@Alg/Cs extracts for 1, 3, and 5 days (*n* = 5). C) CLSM images of Caco‐2 cells after incubation with free FITC and FITC‐PDAP NPs for 4 h. Scale bars, 200 µm. D) Quantification of the relative fluorescence intensity of FITC (*n* = 3). E) Fluorescence images of intestines at different time points after intravenous injection of FITC‐PDAP NPs, oral administration of FITC‐PDAP NPs, or FITC‐PDAP@Alg/Cs. F) Fluorescence images of femurs at different time points after oral administration of FITC‐PDP@Alg/Cs or FITC‐PDAP@Alg/Cs. G) Fluorescence images of organs (heart, liver, spleen, lung, and kidney) at 4 h after gavage of FITC‐PDP@Alg/Cs or FITC‐PDAP@Alg/Cs.

To ensure that the same number of microspheres was co‐cultured with cells, we performed biocompatibility experiments using hydrogel microsphere extracts. After co‐culturing the intestinal epithelial cell line Caco‐2 with various concentrations of hydrogel microsphere extracts for 1, 3, and 5 days, we investigated the biocompatibility of PDAP@Alg/Cs using qualitative and quantitative methods. According to Live/Dead staining data, 25% PDAP@Alg/Cs extracts had a substantial inhibitory effect on cells at days 3 and 5, while Alg/Cs, 1% PDAP@Alg/Cs, and 5% PDAP@Alg/Cs extracts had similar biocompatibility to the control (**Figure** **4**A; and Figure S8, Supporting Information). The 5% PDAP@Alg/Cs extracts retained good biocompatibility at day 5, as reported by cell counting kit‐8 (CCK‐8) data (Figure 4B). The slight cytotoxicity of 25% PDAP@Alg/Cs extracts could be caused by the extremely high drug concentration. Therefore, the following experiments in this study utilized 5% PDAP@Alg/Cs hydrogel microspheres to account for both the drug concentration and biocompatibility.

We then observed drug uptake by Caco‐2 cells using CLSM. Compared to Caco‐2 cells incubated with free FITC, cells incubated with FITC‐PDAP NPs, which were fabricated by FITC‐modified PDAP NPs, displayed a more intense green fluorescent signal at 4 h (Figure 4C,D). As a result, organic–inorganic nanoparticles can significantly increase the efficiency of drug entry into intestinal cells. To observe the intestinal absorption of PDAP@Alg/Cs in mice in vivo, after oral administration of FITC‐PDAP@Alg/Cs hydrogel microspheres, we tracked the in vivo FITC signal intensity visually using the In Vivo Imaging System (IVIS). Compared with tail vein injection or intragastric administration of FITC‐PDAP, the fluorescence signal intensity distribution in the intestine of mice that were orally administered FITC‐PDAP@Alg/Cs hydrogel microspheres was stronger and lasted longer in the intestine, for up to 8 h (Figure 4E). The above results could be attributed to increased intestinal absorption and enhanced retention of PDAP NPs in the intestine. On the other hand, the distribution of PDAP NPs in various mouse tissues was observed by IVIS to test the efficacy of the bone‐targeting drug delivery system. The results demonstrated that compared with intragastric administration of FITC‐PDP@Alg/Cs hydrogel microspheres, lacking the bone‐targeting peptide Asp8 compared to PDAP NPs, the fluorescence signal intensity was higher in the femur of mice administered FITC‐PDAP@Alg/Cs hydrogel microspheres orally, reaching a peak at 4 h, while the fluorescence signal intensity was lower in other organs (Figure 4F,G).

### 2.4 Promotion of Vascularization In Vitro

**Figure 5:**
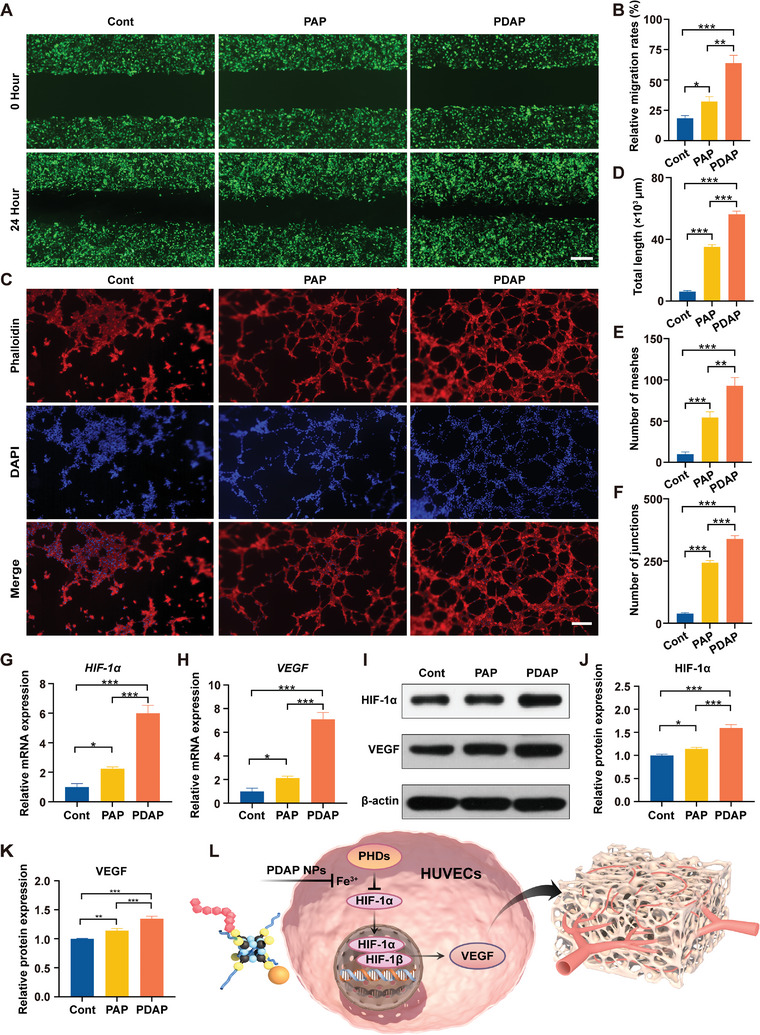
In vitro promotion of vascularization. A) Representative photomicrographs of HUVECs migration after incubation of 0 and 24 h. Scale bars, 400 µm. B) Quantitative analysis of wound migration rate on the cell migration results (*n* = 3). C) Representative photomicrographs of HUVECs tube formation on Matrigel. Scale bars, 50 µm. D) Total length of vascular tube formation (*n* = 3). E) Number of meshes (*n* = 3). F) Number of junctions (*n* = 3). G,H) Quantitative RT‐PCR detection of mRNA expression of *HIF‐1α* and *VEGF* (*n* = 3). I) Representative Western blot results for HIF‐1*α* and VEGF. J–K) Quantitative analysis of protein levels obtained from Western blot (*n* = 3). L) Schematic representation of PDAP NPs promoting vascularization.

To ascertain whether PDAP NPs have a vascularization‐promoting effect in vitro, we used PDAP NPs to intervene in human umbilical vein endothelial cells (HUVECs). Before this assay, we carried out the CCK‐8 assay to assess the effect of PDAP NPs on the activity of HUVECs, and the results revealed that PDAP NPs concentrations up to 50 µm were not significantly toxic to HUVECs (Figure S9, Supporting Information). Subsequently, we employed a cell migration assay and a tube formation test to clarify whether PDAP NPs can promote angiogenesis in vitro. First, we synthesized PAP NPs, which lack DFO compared to PDAP NPs. After 24 h of PDAP NPs intervention on HUVECs, the cell migration experiment revealed that compared to the PAP and control group, the PDAP group dramatically increased the migration of HUVECs (**Figure** **5**A,B). Our assay demonstrated that the tube formation was significantly enhanced in the PDAP group compared with the PAP and control groups (Figure 5C). Similar conclusions were drawn using Image J software analysis of the total length, the number of meshes, and the number of junctions (Figure 5D–F). HIF‐1*α* and VEGF are important regulatory factors in the process of intraosseous angiogenesis.^[^ ^4a^ ^]^ In Figure 5G,H, HIF‐1*α* and VEGF mRNA expression in the PDAP group was considerably greater than in the PAP and control groups, with the PAP group having higher HIF‐1*α* and VEGF mRNA expression than the control group. HIF‐1*α* and VEGF protein expression showed similar results (Figure 5I–K). The aforementioned in vitro investigations indicated that PDAP NPs promoted angiogenesis by activating HIF‐1*α*/VEGF signaling pathway (Figure 5L).

### 2.5 Inhibition of Bone Resorption In Vitro

**Figure 6:**
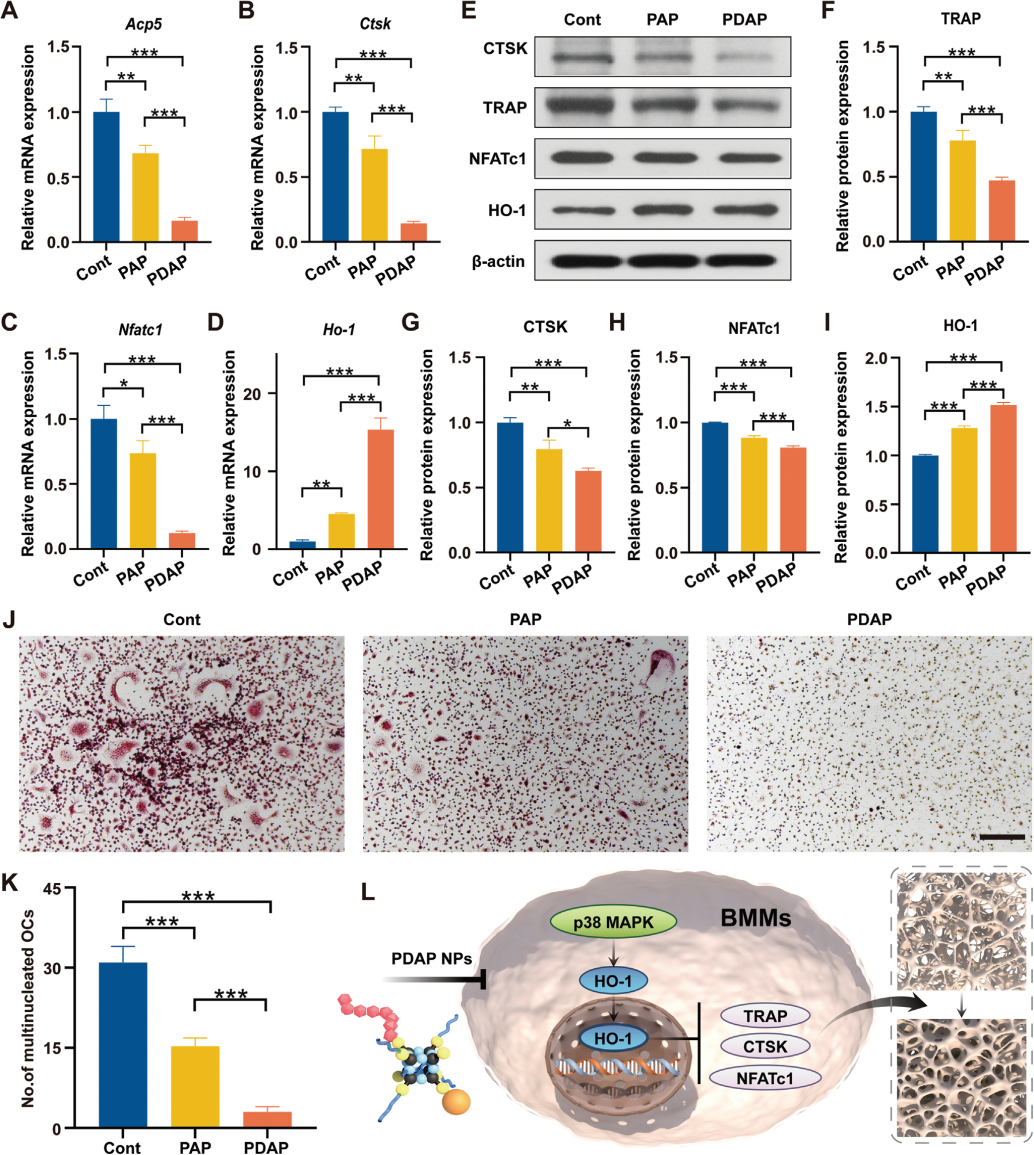
In vitro inhibition of bone resorption. A–D) Quantitative RT‐PCR detection of mRNA expression of osteoclast‐related genes (*Acp5*, *Ctsk*, and *Nfatc1*) and *Ho‐1* in BMMs (*n* = 3). E) Representative Western blot results for osteoclast‐related genes (TRAP, CTSK, and NFATc1) and HO‐1 in BMMs. F–I) Quantitative analysis of protein levels obtained from Western blot (*n* = 3). J) TRAP staining images of BMMs‐derived osteoclasts. Scale bars, 200 µm. K) Quantification analysis of the number of TRAP‐positive cells (*n* = 3). L) Schematic representation of PDAP NPs inhibition of bone resorption.

To exclude the inhibition of osteoclastogenesis due to PDAP NPs toxicity, we employed a CCK‐8 assay to detect the impact of PDAP NPs on the cell viability of bone marrow‐derived mononuclear macrophages (BMMs). The results revealed that PDAP NPs concentrations up to 50 µm were not particularly hazardous to BMMs (Figure S10, Supporting Information). Several genes, including *Acp5* (encoding TRAP), *Ctsk* (encoding Cathepsin K), and *Nfatc1* (encoding NFATc1), are involved in osteoclast differentiation and formation.^[^ ^14^ ^]^ To determine whether PDAP NPs have a suppressive effect on bone resorption in vitro, we detected the impact of PDAP NPs on the expression of these genes. The mRNA expression of *Acp5, Ctsk*, and *Nfatc1* in the PDAP group was considerably lower than in the PAP and control groups, while the mRNA expression of *Acp5, Ctsk*, and *Nfatc1* in the PAP group decreased compared to the control group (**Figure** **6**A–C). The protein expression of TRAP, CTSK, and NFATc1 produced similar results (Figure 6E–H). HO‐1 is a rate‐limiting enzyme for heme degradation, and it has been noted that DFO can suppress osteoclast development by boosting HO‐1 expression. The HO‐1 mRNA and protein expression were considerably higher in the PDAP group, according to quantitative real‐time PCR and Western blot results (Figure 6D–I). Inhibition of osteoclastogenesis in vitro was further clarified by induction of BMMs with 30 ng mL^−1^ macrophage‐colony stimulating factor (M‐CSF) and 75 ng mL^−1^ receptor activator of nuclear factor‐*κ*B ligand (RANKL) for 5 days. Tartrate‐resistant acid phosphatase (TRAP) staining revealed that there were almost no mature osteoclasts in the PDAP group and 50% fewer TRAP‐positive osteoclasts in the PAP group compared to the control group (Figure 6J,K). These data showed that PDAP NPs activated HO‐1 to decrease osteoclast activity and formation (Figure 6L).

### 2.6 Antiosteoporotic Effects in OVX Mice

**Figure 7:**
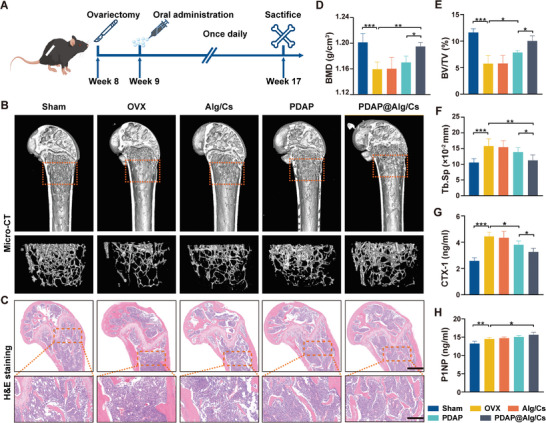
In vivo antiosteoporosis effect in OVX mice. A) Experimental design. Eight‐week‐old C57BL/6 female mice were selected and bilateral ovaries were removed to construct a postmenopausal osteoporosis model. Oral administration was performed once daily after 1 week for 8 weeks, all mice then were euthanized and samples were extracted. B) Representative Micro‐CT images of the distal femur in the Sham, OVX, Alg/Cs, PDAP, and PDAP@Alg/Cs group. C) Representative images of H&E staining of the distal femur in different groups. Scale bars, 500 and 200 µm for top and bottom images, respectively. D) BMD, E) BV/TV, and F) Tb. Sp of the distal femur in different groups from micro‐CT images (*n* = 6). G) Concentration of serum H) CTX‐1 and I) P1NP via ELISA measurement (*n* = 6).

Eight‐week‐old female C57BL/6 mice were selected and the classic postmenopausal osteoporosis (OVX) model was constructed by removing bilateral ovaries. We observed all mice by dividing them into 5 random groups: Sham, OVX, Alg/Cs, PDAP, and PDAP@Alg/Cs group. The first two groups received 1% sodium carboxymethylcellulose orally administered once daily for 8 weeks and were then euthanized (**Figure** **7**A). Micro‐CT and H&E staining of the distal femur revealed that bone trabeculae were severely reduced in the OVX group, demonstrating that the OVX mouse model was successfully constructed (Figure 7B,C). Compared to the OVX and PDAP groups, the number of bone trabeculae was considerably higher in the PDAP@Alg/Cs group (Figure 7C). When compared to the control group, the PDAP group showed increased BMD and bone volume fraction (BV/TV), as well as decreased bone trabeculae (Tb. Sp). Meanwhile, the PDAP@Alg/Cs group showed an even greater increase in BMD and BV/TV, as well as a greater decrease in Tb. Sp (Figure 7D–F). However, PDAP@Alg/Cs did not enhance cortical bone‐related parameters (Figure S11, Supporting Information). The serum enzyme linked immunosorbent assay (ELISA) results also demonstrated that CTX‐1 levels were significantly lower and P1NP levels were much higher in the PDAP@Alg/Cs group (Figure 7G,H). According to the aforementioned findings, PDAP NPs exhibited more pronounced osteoprotective effects after oral administration of PDAP@Alg/Cs because Alg/Cs helped to promote intestinal absorption and protect PDAP NPs from being destroyed in the stomach.

**Figure 8:**
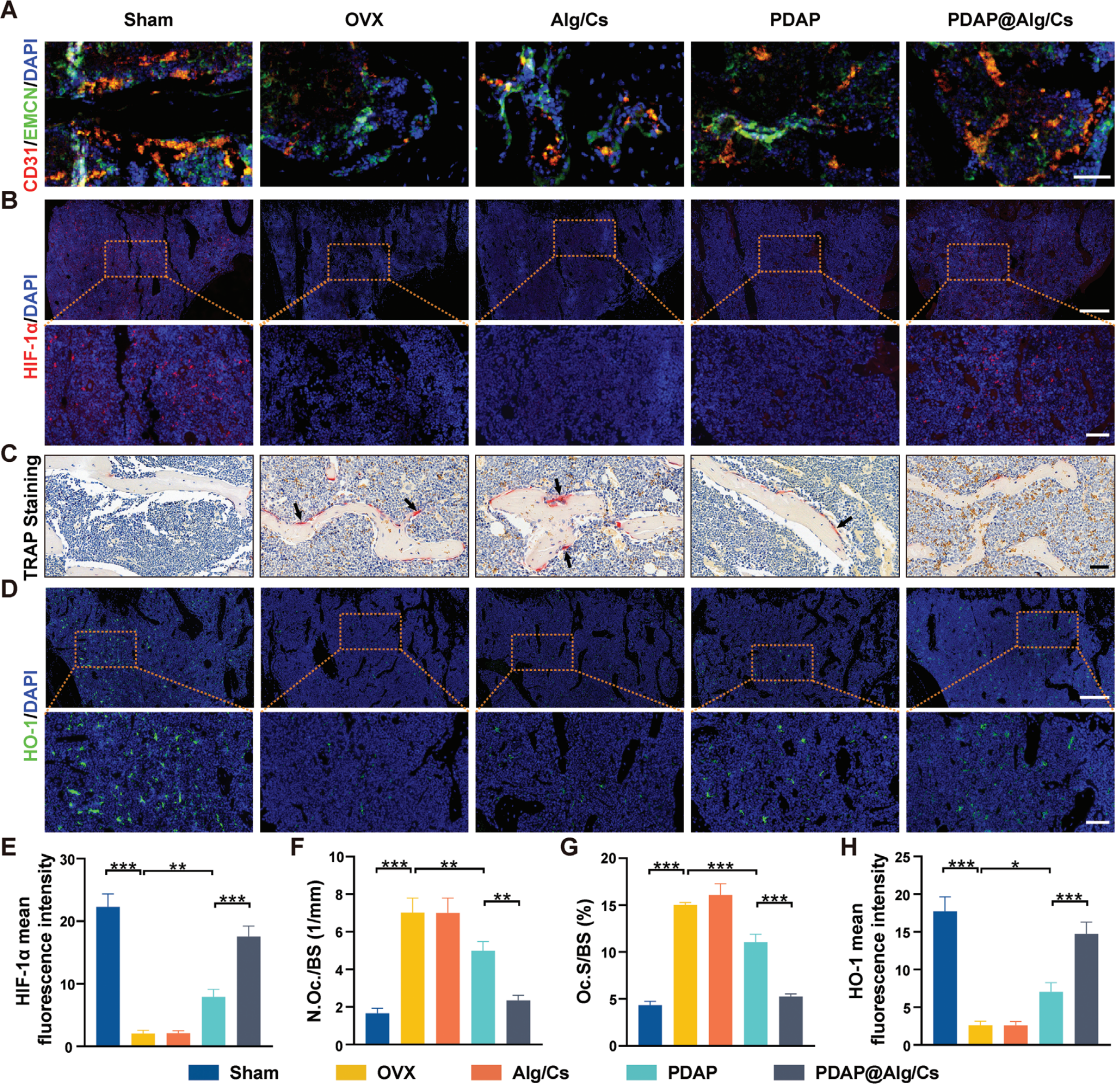
In vivo negative regulation of intraosseous angiogenesis and bone resorption. A) Representative image of CD31^hi^EMCN^hi^ microvessels in the distal femur. Scale bars, 50 µm. B) Immunofluorescence staining of HIF‐1*α* in the distal femur. Scale bars, 200 and 50 µm for top and bottom images, respectively. C) Representative images of TRAP staining of the distal femur. Arrows point to multinucleated osteoclasts. Scale bars, 50 µm. D) Immunofluorescence staining of HIF‐1*α* in the distal femur. E) Scale bars, 200 and 50 µm for top and bottom images, respectively. F) Quantification of the HIF‐1*α* mean fluorescence intensity in different groups (*n* = 6). Quantification of G) No.Oc./B.Pm, H) Oc.S/BS from C) (*n* = 6). I) Quantification of HO‐1 mean fluorescence intensity in different groups (*n* = 6).

Histological sections were employed to identify the mechanism of in vivo antiosteoporotic effects. We employed immunofluorescence to discover Endomucin (EMCN) and CD31 expression. The number of type H vessel (CD31^hi^EMCN^hi^) was dramatically enhanced in the PDAP@Alg/Cs group with a striated distribution, but significantly decreased in the OVX mice with sparse and punctate distribution (**Figure** **8**A). HIF‐1*α* in endothelial cells is a critical factor at the beginning of type H vessel formation in the bones.^[^ ^2b^ ^]^ To verify whether oral PDAP@Alg/Cs could activate the HIF‐1*α* signaling pathway, we used immunofluorescence to identify HIF‐1*α* expression. The PDAP@Alg/Cs group had the highest level of HIF‐1*α* fluorescence intensity except for the Sham group, according to the data (Figure 8B,E). In the PDAP@Alg/Cs group, TRAP staining revealed that the count and percentage of osteoclasts on the bone surface considerably decreased, almost reaching normal levels (Figure 8C,F,G). The fluorescence intensity of the osteoclast marker NFATc1 was also noticeably lower in the PDAP@Alg/Cs group (Figure S12, Supporting Information). Additionally, HO‐1 immunofluorescence was detected and the findings indicated that HO‐1 expression was markedly greater in the PDAP@Alg/Cs group (Figure 8D,H). All these outcomes were in line with those of in vitro experiments.

The major organs (heart, liver, spleen, kidney, and intestine) were observed using H&E staining to further confirm the biocompatibility following oral PDAP@Alg/Cs. No significant pathological changes such as necrosis and inflammation were found in the sections of each organ. According to these findings, oral administration of PDAP@Alg/Cs demonstrated good biocompatibility in vivo (Figure S13, Supporting Information).

## 3 Discussion

To increase the retention time and bioavailability of medicines in the gastrointestinal system, we developed a pH‐responsive micro/nano‐hydrogel microsphere that can be orally administered in this study. We employed the one‐pot method to create organic–inorganic hybrid nanoparticles and a gas microfluidic technique to generate hydrogel microspheres with a shell–core structure. Both of these preparation techniques are quick, easy, and allow for mass production. This innovative micro/nano‐hydrogel microsphere system can considerably enhance the antiosteoporosis impact in the OVX mice model by realizing the negative regulation between intraosseous angiogenesis and bone resorption to prevent bone loss. Additionally, the experiments in vitro and in vivo confirmed that the hydrogel microsphere system had a slow/controlled release in the intestine and actively targeted to bone tissue, which significantly decreased the adverse effects of the drug. Therefore, the oral delivery system for the bone‐targeting micro/nano‐hydrogel microspheres described in this study is a potential therapeutic approach for the management of PMOP, and can also be extended to oral delivery of other drugs for further application in the treatment of other systemic diseases.

It is general knowledge that bone tissue is a highly vascularized connective tissue, accompanied by a huge network of capillaries and large blood tubes.^[^ ^15^ ^]^ The vascular system regulates bone development, regeneration, and remodeling by supplying neurotransmitters, oxygen, hormones, growth factors, and nutrients to bone tissue cells.^[^ ^16^ ^]^ Recently, Kusumbe et al.^[^ ^2^, ^17^ ^]^ revealed that type H vessels, a special capillary subtype with high expression of CD31 and EMCN, are surrounded by clusters of Osterix‐positive osteoprogenitor cells, which couple angiogenesis and bone formation in regions of active bone growth, such as the epiphysis and periosteal regions. Wang et al.^[^ ^4b^ ^]^ found that the abundance of type H vessel in the bones is an early biomarker of bone loss and the amount of type H vessel is equally declined in the elderly and osteoporotic populations. On the other hand, PMOP is a metabolic bone disorder arising from a sharp drop in estrogen levels as a result of declining ovarian function. These lead to a loss of coupling between bone formation and bone resorption, disrupting bone metabolism and causing a reduction in bone mass. As such, existing antiosteoporosis medicines frequently target osteoclasts as their primary intervention target, while ignoring the special function that vascularization plays in bone reconstruction. To address these issues, we grafted DFO and Asp8 onto the POSS nanoplatform as a base material, then constructed a hydrogel microsphere with pH‐responsive properties for oral delivery.

Alginate and chitosan, two natural polysaccharides that have received FDA approval, are frequently utilized for the oral delivery of cells, probiotics, bovine serum albumin, and insulin due to their abundant availability and biocompatibility.^[^ ^18^ ^]^ Chitosan has a positive charge that electrostatically interacts with mucin on the intestinal mucosa. It can open tight junctions between epithelial cells of the intestinal mucosa, extend drug retention time, and increase drug paracellular permeation, all of which improve drug intestinal absorption.^[^ ^19^ ^]^ Due to the poor swelling rate of the microspheres caused by the interaction of the carboxyl groups in alginate and the amino groups in chitosan, the micro/nanogel microspheres generated in this study only released 16% PDAP NPs in SGF. The chelation of carboxyl groups in alginate by phosphate ions caused the microspheres in SIF and SCF to quickly inflate, and the drug release rate reached 80% after 4 h in SIF. The microspheres in SCF with higher pH deteriorated over time, reaching a nearly full state of degradation after 24 h. Furthermore, IVIS imaging showed a stronger distribution of fluorescent signal intensity in the intestine of mice with intragastric administration of PDAP@Alg/Cs hydrogel microspheres for up to 8 h.

A growing body of research suggests that DFO can inhibit prolyl hydroxylase (PHDs) activity, impede pathological iron deposition in bone tissue and activate HIF‐1*α*/VEGF signaling pathway, demonstrating promising therapeutic effects for osteoporosis and bone regeneration.^[^ ^4^, ^20^ ^]^ Nevertheless, the clinical application of DFO for the prevention and treatment of PMOP is severely hampered by the extremely low half‐life of DFO (only ≈5–15 min in rodents), inconvenient administration method, continuous dosing time, and numerous adverse effects caused by high concentrations due to rapid drug absorption. Some studies sought to graft DFO onto biocompatible polymers or nanoparticles to optimize pharmacokinetics/pharmacodynamics and tissue distribution, since patients with iron overload frequently require long‐term drug therapy.^[^ ^21^ ^]^ Park et al.^[^ ^22^ ^]^ prepared an injectable temperature‐sensitive hydrogel loaded with DFO nanoparticles, which greatly prolonged the renal clearance of the medicine after subcutaneous injection. Guo et al.^[^ ^23^ ^]^ generated a bone‐seeking DFO derivative (SF‐DFO) based on DFO and iminodiacetic acid, and administered it intraperitoneally to significantly boost HIF‐1*α* expression and angiogenesis, increase bone density, as well as decrease biotoxicity in OVX mice. In the current study, DFO and Asp8 were cografted onto the POSS nanoplatform to develop hydrogel microsphere systems loaded with PDAP NPs, which retained DFO activity in vitro and in vivo, exhibited a slow/controlled release in the intestines, and targeted the bones. IVIS demonstrated that PDAP NPs grafted with Asp8 had more intense and persistent fluorescent signals in bone. This also supports previous reports that liposomes containing eight aspartate residues (Asp8) can effectively target bone resorption surfaces for site‐specific drug delivery and effective mitigation of bone loss.^[^ ^24^ ^]^ Mice administered with PDAP@Alg/Cs hydrogel microspheres orally had increased levels of vascularization and bone mass, according to micro‐CT and histological sections. In conclusion, our research indicates that PDAP NPs can successfully deliver DFO to bone tissue, exert antiosteoporosis benefits and lessen adverse medicine reactions.

On the other hand, Xie et al.^[^ ^3a^ ^]^ found that PDGF‐BB excreted by pro‐osteoclasts could induce the formation of type H vessel, enhancing the coupling between angiogenesis and bone formation. Therefore, there could be a negative coupling between intraosseous angiogenesis and bone resorption during bone remodeling. Huang et al.^[^ ^25^ ^]^ reported that Harmine inhibited RANKL‐induced multinucleated osteoclast formation and alleviated bone resorption while inducing type H vessel formation. Likewise, Song et al.^[^ ^26^ ^]^ reported that Nuciferine suppressed the formation of RANKL‐induced multinucleated osteoclasts by inhibiting mitogen‐activated protein kinase (MAPK) and nuclear factor‐𝜅B (NF‐𝜅B) signaling pathways, preventing the production of multinucleated osteoclasts and increasing type H vessel formation. Intriguingly, DFO inhibits osteoclastogenesis by upregulating HO‐1 via the p38 MAPK signaling pathway.^[^ ^27^ ^]^ HO‐1, an intracellular oxidative stress regulator, catalyzes the production of biliverdin, carbon monoxide, and free ferrous iron from heme, which exerts protective effects against various human diseases, such as cardiovascular disease, neurodegenerative disease, and diabetes.^[^ ^28^ ^]^ It has been reported that HO‐1^−/−^ mice have lowered bone mass along with increased osteoclast number and activity, high serum TRAP levels, and that HO‐1 maintains bone mass by negatively influencing osteoclasts. Therefore, promoting HO‐1 expression could be a potential therapeutic target for treating osteoporosis.^[^ ^29^ ^]^ Our research suggests that DFO‐loaded PDAP NPs can enhance HO‐1 expression while suppressing osteoclast‐related gene expression such as *Acp5*, *Nfatc1*, and *Ctsk*, as well as osteoclast activity.

Strikingly, this study revealed that PAP NPs unloaded with DFO had a vascularization‐promoting and resorption‐inhibitory effect, which could be relevant to the release of silica ions upon their degradation.^[^ ^30^ ^]^ According to earlier research, novel hydrogels made from POSS and gelatin promote the expression of integrin *α5β1* and significantly enhance the adhesion and proliferation of bone marrow mesenchymal stem cells (BMSCs) and HUVECs, with positive effects on facilitating angiogenesis and bone regeneration.^[^ ^31^ ^]^ Hence, POSS nanoparticles, acting as carriers, not only can sustainably deliver DFO, but improve the regulation of vascularization and bone metabolism itself.

Although the micro/nano‐hydrogel microsphere system developed in this study for oral delivery, negatively affecting intraosseous angiogenesis and bone resorption, is a promising and safe antiosteoporosis drug, the following points still need to be explored in future research: 1) more comprehensive pharmacokinetic/pharmacodynamic studies to understand the in vivo metabolism and distribution of the drug; 2) development of “diagnosis and treatment integration” biomedical materials by linking biofluorescent probes to the eight modification sites on POSS; 3) comparison of the therapeutic effects of PDAP@Alg/Cs with the most widely used clinical anti‐osteoporosis drugs, such as bisphosphonates and denosumab; 4) safety assessment in large animals or nonhuman primate models to provide a theoretical basis for clinical translation.

In conclusion, we used an innovative POSS nanoplatform to efficiently synthesize organic–inorganic hybrid nanoparticles (PDAP NPs) via click chemistry and successfully developed a pH‐responsive micro/nano‐hydrogel microsphere system encapsulating PDAP NPs via gas microfluidic and ionic cross‐linking techniques. These can achieve oral delivery, intragastric protection, slow/controlled release in the intestine, and active targeting of bone tissue. In vitro experiments showed that PDAP@Alg/Cs released just 16% in SGF and up to 80% in SIF. This hydrogel microsphere system not only adheres to the intestine and effectively improves the drug intestinal absorption rate and bioavailability, but also enables targeted delivery to the bone‐resorbing surface. Additionally, we demonstrated through in vivo tests that PDAP@Alg/Cs markedly reduced serum CTX‐1 levels, decreased the number of TRAP‐positive osteoclasts, promoted the formation of type H vessels in bone, and effectively alleviated bone loss. Therefore, our study provides the first in‐depth study of the oral DFO delivery system to negatively affect intraosseous angiogenesis and bone resorption at both in vitro and in vivo levels, offering a safe and effective approach of oral administration to prevent and treat PMOP.

## 4 Experimental Section

### Materials

3‐mercaptopropyltriethoxysilane, acryloyl chloride, polyethylene glycol 400, tetrahydrofuran (THF), and potassium bromide (KBr) were all purchased from Sinopharm Chemical Reagent Co., Ltd. (Shanghai, China). DFO and Asp8 were purchased from Sigma‐Aldrich (St. Louis, MO) and China Peptides Co., Ltd. (Shanghai, China), respectively. Caco‐2 and HUVECs cells were obtained from the Cell Bank of Chinese Academy of Sciences (Shanghai, China). POSS‐SH and acryloyl chloride‐modified polyethylene glycol 400 (PEG‐Ac) have been synthesized in the laboratory.^[^ ^32^ ^]^

### Synthesis of PDAP NPs

0.363 mg of PEG‐Ac (0.8 mmol), 0.015 g of 2,2‐dimethoxy‐2‐phenylacetophenone (DMPA) (0.06 mmol), and 2.032 g of POSS‐SH (0.2 mmol) were added into 20.0 mL of THF to react for 2.5 h under 365 nm UV light with N_2_ protection at room temperature. Then, this reaction solution was mixed with 10.00 mL of THF containing 0.1200 g of DMPA (0.47 mmol), 0.0970 g of Asp8‐Ac (0.1 mmol), and 0.0320 g of DFO‐Ac (0.05 mmol) and continued to react for 8 h (Figure S14, Supporting Information). Finally, the solvent was removed by rotation. Yield: 91.3%.

FTIR (KBr): *v* = 3434 cm^−1^ (OH); 3350 cm^−1^, 3279 cm^−1^ (NH); 1710 cm^−1^ (C=O); 1659 cm^−1^ (C=N); 3055 cm^−1^, 1689 cm^−^1, 1531 cm^−1^, 1492 cm^−1^ (C_6_H_6_); 1276 cm^−1^ (C—O). 1H NMR (CDCl_3_, 600 MHz, ppm): *δ* = 0.71 (d, Si—CH_2_—), 4.19 (s, OH),2.48 (s, —CH_2_—S),1.48 (—SH), 1.59 (s, —CH_2_—), 6.28, 6.95–8.11(d, ArH),3.61 (s, CH_2_—OH), 10.83–10.29 (d, —NH).

PDP NPs and PAP NPs were synthesized without the addition of Asp8‐Ac and DFO‐Ac reagents, respectively.

### Characterization of PDAP NPs

^1^H NMR spectra were detected with Bruker AMX‐600 NMR spectrometer (Bruker, Switzerland) at 600 MHz. FTIR spectra were recorded with Thermo Nicolet 8700 infrared spectrometer (Thermo Fisher, Waltham, MA) using KBr powder. UV–Vis and fluorescence spectra were detected by Lambda‐35 spectrometer (PerkinElmer, Inc., Shelton, CT) and LS‐55 fluorescence spectrometer (PerkinElmer, Inc., Shelton, CT), respectively. The morphology of PDAP NPs was observed by transmission electron microscopy (Hitachi, Japan), and the molecular weight of PDAP NPs was determined with a MALDI‐TOF mass spectrometer (Bruker, Billerica, MA). The HOMO–LUMO orbital energy levels were calculated at the B3LYP/6‐31G/LANL2DZ level using density functional theory (DFT) in Gaussian 03 software. The DFT‐based B3LYP/6‐31g(d) model is applicable to molecular systems with C, N, and O atoms.

### Synthesis of Hydrogel Microspheres

Hydrogel microspheres were prepared using a gas microfluidic technique.^[^ ^33^ ^]^ Many factors affect the quality of hydrogel microspheres, such as nitrogen flow, flow rate of sodium alginate, sodium alginate concentration, receiving angle of coaxial needle, and receiving distance (distance between the tip of the coaxial needle and the collecting dish containing the calcium chloride solution). In the preparation process, it was first ensured that the sodium alginate solution was 1% w/v because a higher concentration could easily lead to needle clogging. A nitrogen flow rate of 1.0 L min^−1^ was used, on which the microspheres had excellent morphology and uniform particle size. The sodium alginate flow rate was kept at 15–20 mL h^−1^. The coaxial needle was perpendicular to the ground and the receiving distance should be greater than 9 cm as far as possible. Briefly, a coaxial needle was used to inject sodium alginate solution, which was then sheared into homogenous droplets by the force of nitrogen flow. Then, the droplets were dropped into a calcium chloride (CaCl_2_) solution (100 mm) by gravity. Alg was crosslinked by Ca^2+^, resulting in the formation of the core of hydrogel microspheres. The Alg hydrogel microspheres were collected and the remaining ions on the surface were washed twice with ddH_2_O. They were then immersed in chitosan solution (1%, w/v) and shaken constantly for 30 min (100 rpm, 37 °C). Since the carboxyl group of Alg has a negative charge and the amino group of Cs has a positive charge, the two combined to form a water‐insoluble polymeric electrolyte complex, generating a protective shell of Cs on the surface of Alg hydrogel microspheres. The Alg/Cs hydrogel microspheres were collected and rinsed three times with ddH_2_O to remove the redundant Cs solution, and stored at 4 °C away from light. To prepare PDAP@Alg/Cs, PDAP NPs were dissolved in sodium alginate solution and sonicated for 30 min to form a homogeneously dispersed PDAP/Alg compound, and the remaining steps were performed as aforementioned.

### Characterization of Hydrogel Microspheres

The morphology of the microspheres was observed using an optical microscope (LSM800, ZEISS, Germany) and the diameter of the microspheres was detected using Image J. The zeta potential of the microsphere was measured using dynamic light scattering (DLS) (Zetasizer Nano S, Malvern, UK). The surface morphology of the lyophilized microspheres was observed using a scanning electron microscope (GeminiSEM 300, ZEISS, Germany). To facilitate the observation of the shell–core structure of the microspheres, FITC‐modified chitosan and Rho B‐modified alginate were used to observe the core–shell structure of the microspheres under CLSM (Carl Zeiss, Germany).

### Swelling and Degradation Experiments

The swelling behavior of microspheres was investigated by measuring their water absorption properties using the immersion method. Briefly, 10 mg of freeze‐dried PDAP@Alg/Cs microspheres were put into a 5 mL centrifuge tube and weighed (M_1_). Then, 2 mL of SGF, SIF, and SCF were added, and the mixture was shaken constantly (100 rpm, 37 °C). At the corresponding time point, the supernatant was removed after centrifugation and the redundant liquid was wiped up with filter paper and weighed (M*_t_*). The swelling degree of microspheres was calculated by the following formula

(1) $$\mathrm{Swelling}\;\mathrm{degree}\left(\%\right)=\left[\left(M_{t}-M_{1}\right)/M_{0}\right]\times 100$$

Where M_0_ is the initial mass of microspheres.

The microspheres were put into the simulated gastric and intestinal fluid to examine their degradability. In brief, PDAP@Alg/Cs microspheres were suspended in 2 mL of SGF, SIF, and SCF, and placed on a shaker (100 rpm, 37 °C). The morphological change of the microspheres was observed under an optical microscope at the corresponding time point.

### In Vitro Release Study

To test the release behavior of PDAP NPs, the following techniques were applied. Briefly, 100 mg PDAP@Alg/Cs microspheres were immersed in a dialysis bag containing 10 mL of SGF (containing 0.1% Tween 80) (MW 3500 Da), after which the bag was immersed in 40 mL of the same release buffer and shaken constantly (100 rpm, 37 °C). Then, 2 mL of external release buffer was collected every 0.5 h, while the same volume of buffer was replenished. After 2 h, the microspheres were sequentially transferred to SIF (containing 0.1% Tween 80) and SCF (containing 0.1% Tween 80), and the buffer was collected every 1 h. The concentration of PDAP NPs in each sample was measured using a UV spectrophotometer (Eppendorf, Germany) and release curves were plotted.

The release behavior of PDAP NPs in SGF, SIF, and SCF, respectively, was also examined. The concentration of PDAP NPs in each sample at 1, 2, 4, 6, 8, 10, 12, and 24 h was sequentially measured, then the 24 h release curves were plotted.

### Biocompatibility Evaluation

Caco‐2 cells were selected to study the biocompatibility of microspheres. Briefly, Caco‐2 cells were seeded in 24‐well plates (1×10^4^ cells per well) and cocultured with the various microsphere extracts at 37 °C under 5% CO_2_, similar to the previous reports.^[^ ^34^ ^]^ On days 1, 3, and 5, the cells were incubated with Calcein AM/PI buffer (Proteintech Co., Ltd., China) for 15 min and subsequently observed under a fluorescent microscope (Nikon ECLIPSE Ts2R, Japan). Caco‐2 cells were seeded in 96‐well plates (1 × 10^4^ cells per well) and cocultured with the various microsphere extracts at 37 °C under 5% CO_2_. After cultivation for 1, 3, and 5 days, it was incubated for 1 h in the medium containing a 10% CCK‐8 (Dojindo, Japan) before the absorbance measurement at 450 nm by an enzyme marker (Molecular Devices, Japan).

BMMs and HUVECs were used to investigate the biocompatibility of PDAP NPs. BMMs or HUVECs were seeded in 96‐well plates (1×10^4^ cells per well) and coculture with the different concentrations of PDAP NPs (0, 12.5, 25, and 50 µm) at 37 °C under 5% CO_2_. After cultivation for 48 h, the medium containing 10% CCK‐8 was added and further incubated it for 1 h, and detected its absorbance at 450 nm by enzyme marker.

### Cellular Uptake Analysis

The uptake of PDAP NPs by Caco‐2 cells was observed using CLSM. Briefly, Caco‐2 cells were seeded in 24‐well confocal plates and cocultured with a serum‐free DMEM medium containing the same concentration of FITC or FITC‐PDAP NPs (40 µm) for 4 h. Then, pre‐chilled phosphate buffered saline (PBS) was used to wash Caco‐2 cells three times, followed by using 4% paraformaldehyde (PFA) to fix it for 20 min, and nuclear stain 4′,6‐diamidino‐2‐phenylindole (DAPI, Life Technologies Inc., Gaithersburg, MD) were used to perform nuclear staining for 5 min. Finally, Caco‐2 cells were observed with CLSM.

### IVIS and Tissue Distribution

To facilitate the observation of drug distribution in vivo, the bone‐targeting delivery system (PDAP NPs) and the nontargeting delivery system (PDP NPs) with FITC was modified. Eight‐week‐old female C57BL/6 mice were selected as animal models for tissue imaging. Briefly, tail vein injection of FITC‐PDAP NPs (20 mg kg^−1^ of DFO), gavage of FITC‐PDAP NPs (20 mg kg^−1^ of DFO), or gavage of FITC‐PDAP@Alg/Cs (20 mg kg^−1^ of DFO) was taken. At 1, 2, 4, and 8 h, the intestines of mice were collected after anesthesia and observed using the IVIS (PerkinElmer, Inc., Shelton, CT). Meanwhile, gavage of FITC‐PDP@Alg/Cs (20 mg kg^−1^ of DFO) or FITC‐PDAP@Alg/Cs (20 mg kg^−1^ of DFO) was taken. At 1, 2, 4, 8, and 16 h, the femurs of mice were collected after anesthesia and the hearts, livers, spleens, lungs, and kidneys of mice at 4 h were further collected for assessing drug distribution in vivo.

### Bone Marrow‐Derived Mononuclear Macrophage Extraction

BMMs were separated from the long bones of 8‐week‐old female C57BL/6 mice. Briefly, the marrow suspensions from both the femur and tibia were collected, filtered, and cultured with a complete *α*‐MEM medium containing 30 ng mL^−1^ M‐CSF (R&D Systems, Minneapolis, MN). After 24 h, the cell suspension was collected, centrifuged, and continued to be cultured with a complete *α*‐MEM medium in the presence of 30 ng mL^−1^ M‐CSF. After 3 days, the adherent cells were harvested as precursor osteoclasts.

### Staining of Tartrate‐Resistant Acid Phosphatase (TRAP)

The above‐extracted BMMs were seeded in 24‐well plates (1×10^5^ cells per well) in a complete *α*‐MEM medium containing M‐CSF (30 ng mL^−1^) and RANKL (75 ng mL^−1^) (R&D Systems, Minneapolis, MN) with 50 µm PAP or PDAP NPs and cocultured for 5 days until mature osteoclasts developed. After washing them twice with PBS, the cells were fixed for 15 min with 4% PFA, permeabilized with 0.2% Triton X‐100, and stained with TRAP staining (Servicebio Co., Ltd., China). The number of multinucleated giant cells with positive TRAP and containing three or more nuclei was counted.

### In Vitro Cell Migration Assay

HUVECs expressing green fluorescent protein (GFP‐HUVECs) were seeded in 24‐well plates (1×10^5^ cells per well). A linear wound was generated on the monolayer cells by scratching with a 200 µL sterile pipette tip and the suspended cells were washed with PBS. The scratches were observed by fluorescence microscopy after incubation of 0 and 24 h, and statistical analysis was performed using Image J software.

### In Vitro Tube Formation Assessment

HUVECs were seeded in Matrigel‐coated (Corning Inc., Corning, NY) 24‐well confocal plates (3×10^4^ cells per well) and the cells were incubated in a DMEM medium containing 200 µm PAP or PDAP NPs for 4 h. Then, the cells were fixed with 4% PFA for 20 min, and the Phalloidin (Proteintech Co., Ltd., China) and DAPI were used to label the cytoskeleton and nuclei, respectively. Tube formation was observed by fluorescence microscopy and statistical analysis was performed using Image J software.

### Real‐Time Quantitative PCR

BMMs and HUVECs were seeded in 6‐well plates (1×10^5^ cells per well) and treated with a medium containing 50 µm PAP or PDAP NPs. The extract of total RNA and the obtain of cDNA were performed with TRIzol reagent (Invitrogen Co., Ltd., Grand Island, NY) and PrimeScript Reverse Transcriptase Kit (Takara Bio Inc., Japan), respectively, per the manufacturer's instructions. Real‐time PCR was carried out with SYBR Premix Ex Taq (Takara Bio Inc., Japan). The sequences of primers used are detailed in Table S1 (Supporting Information).

### Western Blot Assays

BMMs and HUVECs were seeded in 6‐well plates (1×10^5^ cells per well) and treated with a medium containing 50 µm PAP or PDAP NPs. The cells were lysed on ice for 0.5 h using RIPA lysate (Servicebio Co., Ltd., China) containing protease inhibitors and the supernatant was collected by centrifugation. After the supernatants were separated by SDS‐PAGE gel electrophoresis, they were transferred to PVDF membranes (Millipore, Billerica, MA) which were then blocked for 1 h with 5% BSA (Servicebio Co., Ltd., China) at room temperature and incubated overnight at 4 °C with the primary antibodies, including anti‐HIF‐1*α* (Servicebio Co. Ltd., China), anti‐VEGF (Servicebio Co., Ltd., China), anti‐TRAP (Abcam, UK), anti‐CTSK (Abcam, UK), anti‐NFATc1 (Cell Signaling Technology, Beverly, MA), anti‐HO‐1 (Servicebio Co., Ltd., China), and anti‐*β*‐actin (Servicebio Co. Ltd., China). After incubation with the corresponding secondary antibodies (Servicebio Co., Ltd., China) for 1 h, the blots were detected by a chemiluminescence system and the optical density values were measured with Image J software.

### Establishment of Osteoporosis Model and Drug Administration

The animal experiments were approved by the Ethics Committee of Soochow University (SUDA20200424A04). Each six random C57BL/6 mice were put into one group. Bilateral ovaries were removed to construct a postmenopausal osteoporosis model, while only bilateral periovarian fat was removed in the Sham group. All mice were raised in a specific pathogen‐free (SPF) environment. All mice received the DFO inject volumes based on the fixed dose (20 mg kg^−1^) of DFO and the different body weights of individuals. The weight of PDAP NPs and PDAP@Alg/Cs according to the reactant proportion of DFO and POSS, and the concentration of PDAP@Alg/Cs was estimated. After a week of rest, the mice were treated with oral administration of Alg/Cs, PDAP NPs, or PDAP@Alg/Cs, which was diluted in 1% sodium carboxymethylcellulose (Aladdin, China), once daily for 8 weeks. The Sham and OVX groups received the same volume of 1% sodium carboxymethylcellulose. Mice serum was separated from their blood by 20 min centrifuged at 3000 rpm at 4 °C. The bilateral femur and tibia, as well as the heart, liver, spleen, kidney, and intestine, were dissected for further analysis.

### Micro‐CT Scanning

Micro‐CT analysis of femur samples was performed to assess the antiosteoporosis effect of PDAP@Alg/Cs microspheres in vivo. The femurs were fixed in 4% PFA and placed in Micro‐CT (Skyscan Co., Belgium) sample inspection plate for scanning and 3D reconstruction (resolution: 9 µm, voltage: 50 kV, source current: 500 mA, rotation step: 0.5°). The region of interest (ROI) of cortical bone in the middle femur and cancellous bone in the distal femur were selected. The following bone morphological parameters were measured and compared: bone density (BMD, g cm^−3^), bone volume/tissue volume (BV/TV, %), trabecular separation (Tb. Sp, mm), cortical bone density (Ct. BMD, g cm^−3^), and cortical thickness (Ct. Th, mm).

### ELISA Assays

Serum levels of CTX‐1 and P1NP were measured using CTX‐1 (Cloud‐Clone Co., Ltd., China) and P1NP (Cloud‐Clone Co., Ltd., China) ELISA kits. All operations were performed according to the manufacturer's instructions.

### Histological and Immunofluorescence Analysis

Bone specimens were fixed in 4% PFA for 48 h and transferred to 10% ethylenediaminetetraacetic acid (EDTA) solution for decalcification for 4 weeks. The solution was changed every other day. They were sequentially dehydrated, paraffin‐embedded, sectioned (5 µm thick), and subjected to H&E staining and TRAP staining. For immunofluorescence staining, block the sections first for 0.5 h with 5% BSA at room temperature and then incubated them overnight at 4 °C with the corresponding primary antibodies: anti‐EMCN (Santa Cruz biotechnology, Santa Cruz, CA), anti‐CD31 (Servicebio Co., Ltd., China), anti‐HIF‐1*α*, anti‐HO‐1, and anti‐NFATc1. After that, they were treated with corresponding secondary antibodies (Servicebio Co., Ltd., China) for 1 h. A fluorescence microscope was employed to obtain images. Image J software was used to analyze the images.

### Statistical Analysis

Statistical data were analyzed via GraphPad Prism 9.0 and SPSS software version 26.0. All data are shown as mean ± SD. The significance between two groups and >3 groups were checked by Student's *t*‐test and one‐way analysis of variance (ANOVA), respectively, with *p < 0.05, **p < 0.01, and ***p < 0.001. NS represents not significant.

## Conflict of Interest

The authors declare no conflict of interest.

## Author Contributions

J.L., G.W., and G.L. contributed equally to this work. J.L. prepared materials, designed, and performed experiments, analyzed data, and wrote the manuscript; G.W. prepared materials and analyzed data; G.L. and R.Z. performed experiments in vitro; Y.D. designed the research and performed experiments in vivo; A.W. and B.L. conceptualized and designed the research; W.C. supervised the research, discussed, revised the manuscript and provided the funding; P.J. and Y.X. designed the research and provided the funding.

## Supporting information

Supporting Information

## Data Availability

The data that support the findings of this study are available from the corresponding author upon reasonable request.
