# Supplementary material for: Regulating Type H Vessel Formation and Bone Metabolism via Bone‐Targeting Oral Micro/Nano‐Hydrogel Microspheres to Prevent Bone Loss
Source: Adv Sci (Weinh). 2023 Mar 26;10(15):2207381. doi: 10.1002/advs.202207381 (PMC10214236; doi:10.1002/advs.202207381)
Supplement: Supplementary file 1 — Supporting Information [file ADVS-10-2207381-s001.pdf]

ADVANCED SCIENCE
Open Access

Supporting Information

for *Adv. Sci.*, DOI 10.1002/advs.202207381

Regulating Type H Vessel Formation and Bone Metabolism via Bone-Targeting Oral Micro/Nano-Hydrogel Microspheres to Prevent Bone Loss

Junjie Li, Gang Wei, Gongwen Liu, Yawei Du, Ruizhi Zhang, Aifei Wang, Baoshan Liu, Wenguo Cui, Peng Jia* and Youjia Xu**

Supplementary Materials for

Regulating type H vessel formation and bone metabolism via bone-targeted oral micro/nano hydrogel microspheres to prevent bone loss

Junjie Li[1,2,4†], Gang Wei[2†], Gongwen Liu[3†], Yawei Du[2], Ruizhi Zhang[1], Aifei Wang[1], Baoshan Liu[1], Wenguo Cui[2*], Peng Jia1[*], Youjia Xu1[*]

*Corresponding author. Email: xuyoujia@suda.edu.cn (Y.X.); jiapengorthop@163.com (P.J.); wgcui80@hotmail.com (W.C.)

This PDF file includes:

Figs. S1 to S14

Tables S1

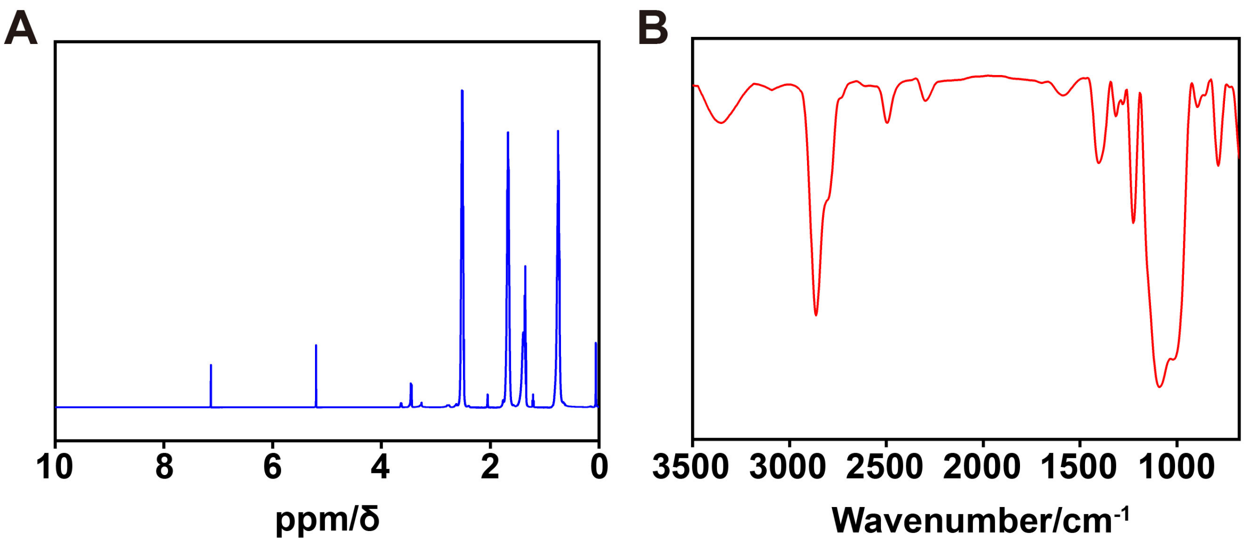

Fig. S1. Characteristics of POSS-SH. (A) ^1H NMR spectra of POSS-SH. (B) FTIR spectra of POSS-SH.

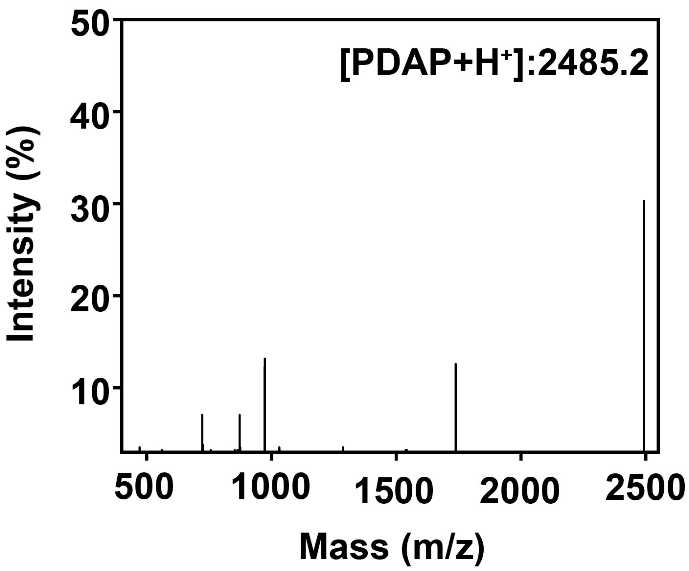

Fig. S2. Molecular weight of PDAP NPs determined by MALDI-TOF.

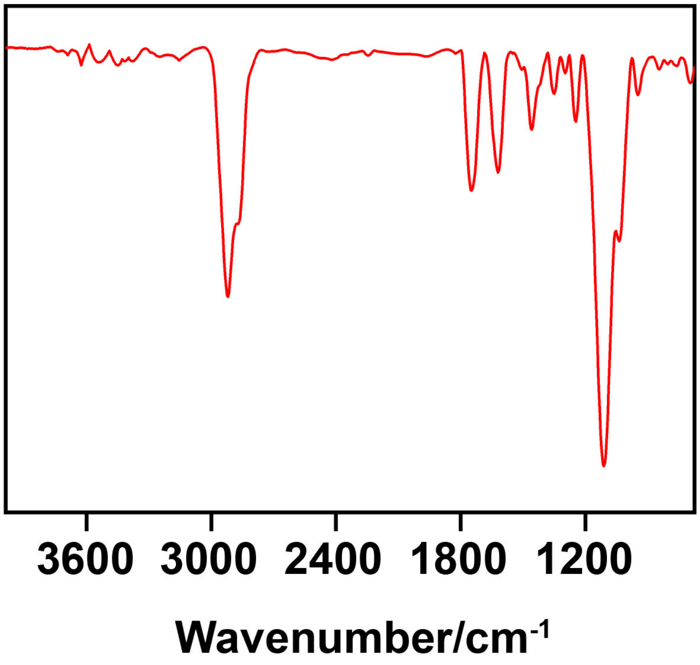

Fig. S3. FTIR spectra of PDAP NPs.

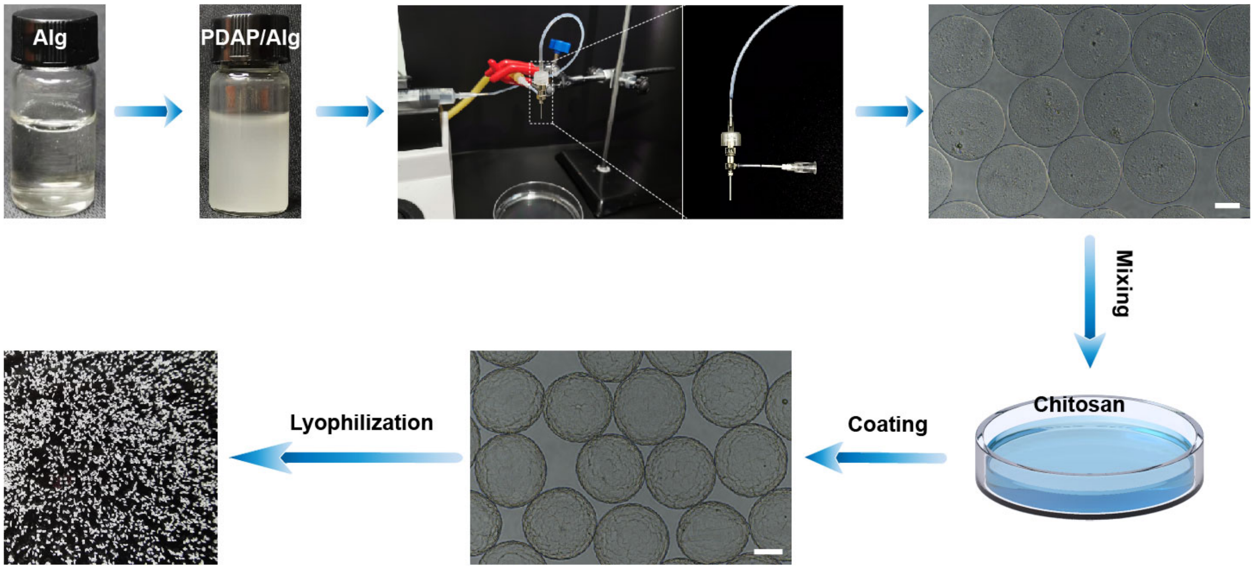

Fig. S4. Preparation process of PDAP@Alg/Cs microspheres *via* gas microfluidic and ionic cross-linking techniques. Scale bars, 100 μm.

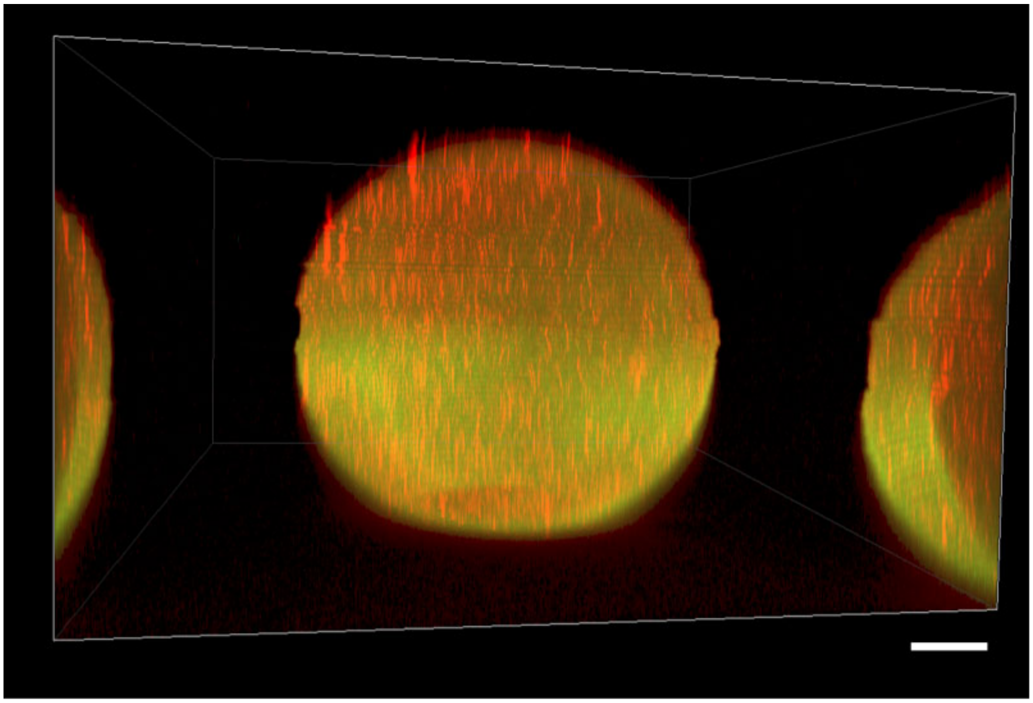

Fig. S5. CLSM 3D image of the shell-core structure of Alg/Cs microspheres. Scale bars, 50 μm.

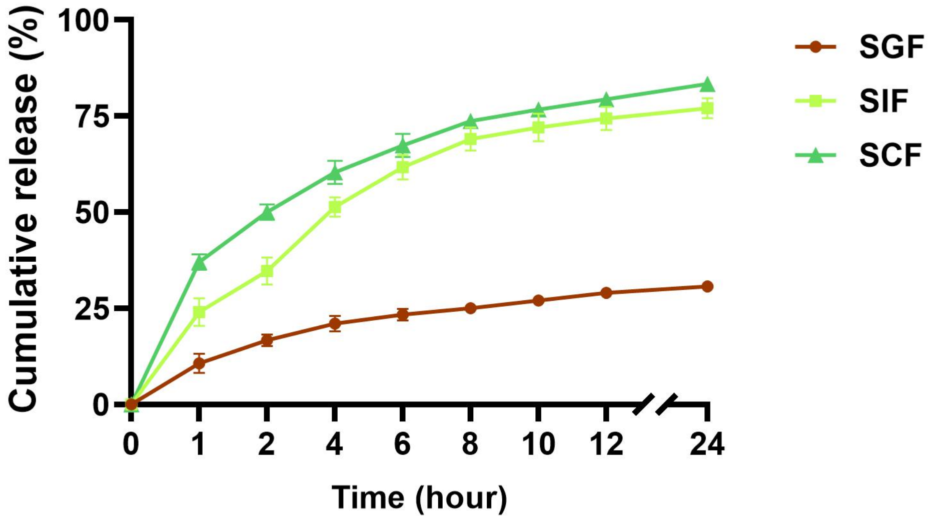

Fig. S6. 24 h release curves of PDAP NPs from PDAP@Alg/Cs microspheres in SGF, SIF, and SCF.

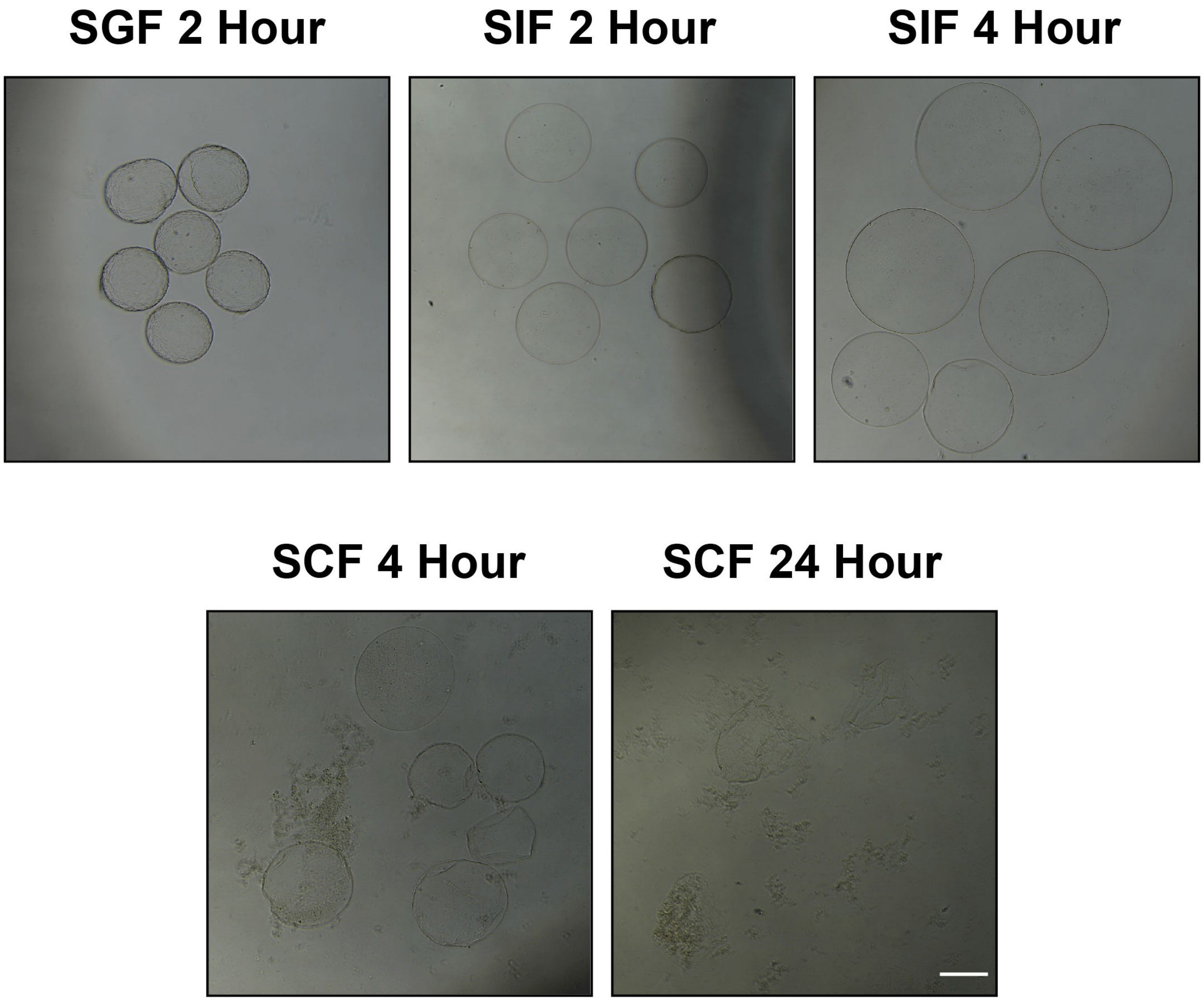

Fig. S7. Optical microscopy images of morphological change of the PDAP@Alg/Cs microspheres in SGF, SIF, and SCF. Scale bars, 200 μm.

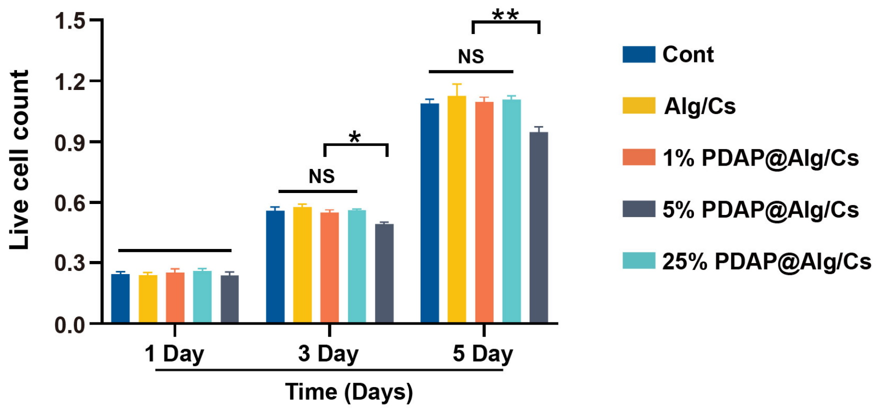

Fig. S8. Live cell count acquired from the Live/Dead staining assay (n=3).

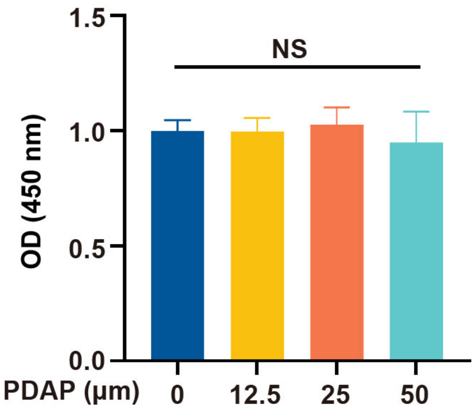

Fig. S9. CCK-8 assay detected the effect of different concentrations of PDAP NPs on HUVECs viability (n=5).

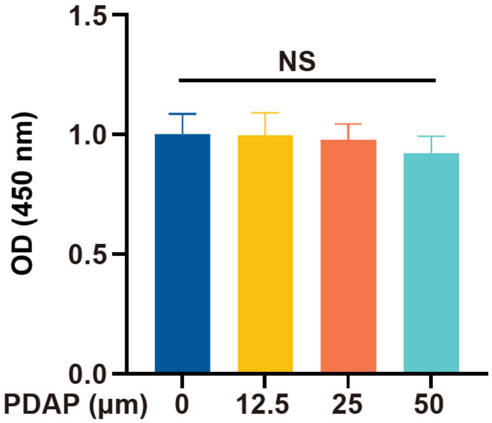

Fig. S10. CCK-8 assay detected the effect of different concentrations of PDAP NPs on BMMs viability (n=5).

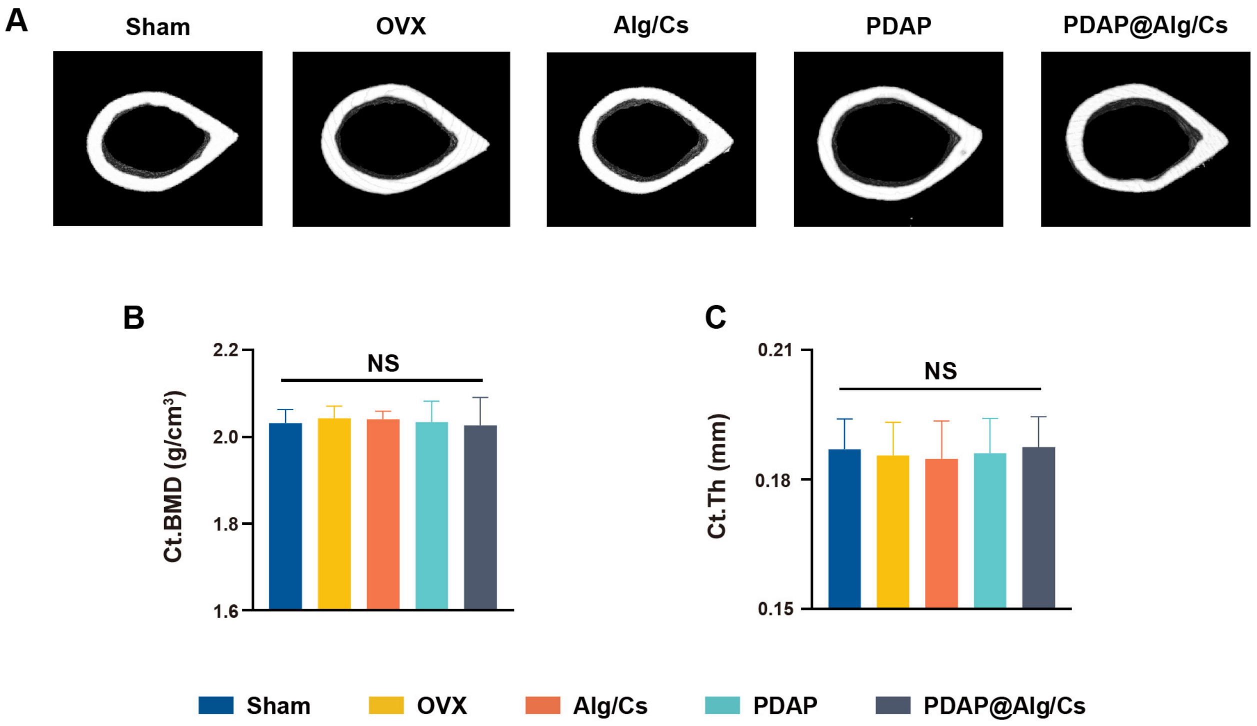

Fig. S11. (A) Representative micro-CT images of the cortical bone of the middle femur in Sham, OVX, Alg/Cs, PDAP, and PDAP@Alg/Cs group. (B) Ct. BMD and (C) Ct. Th of the cortical bone of the middle femur in different groups (n=6).

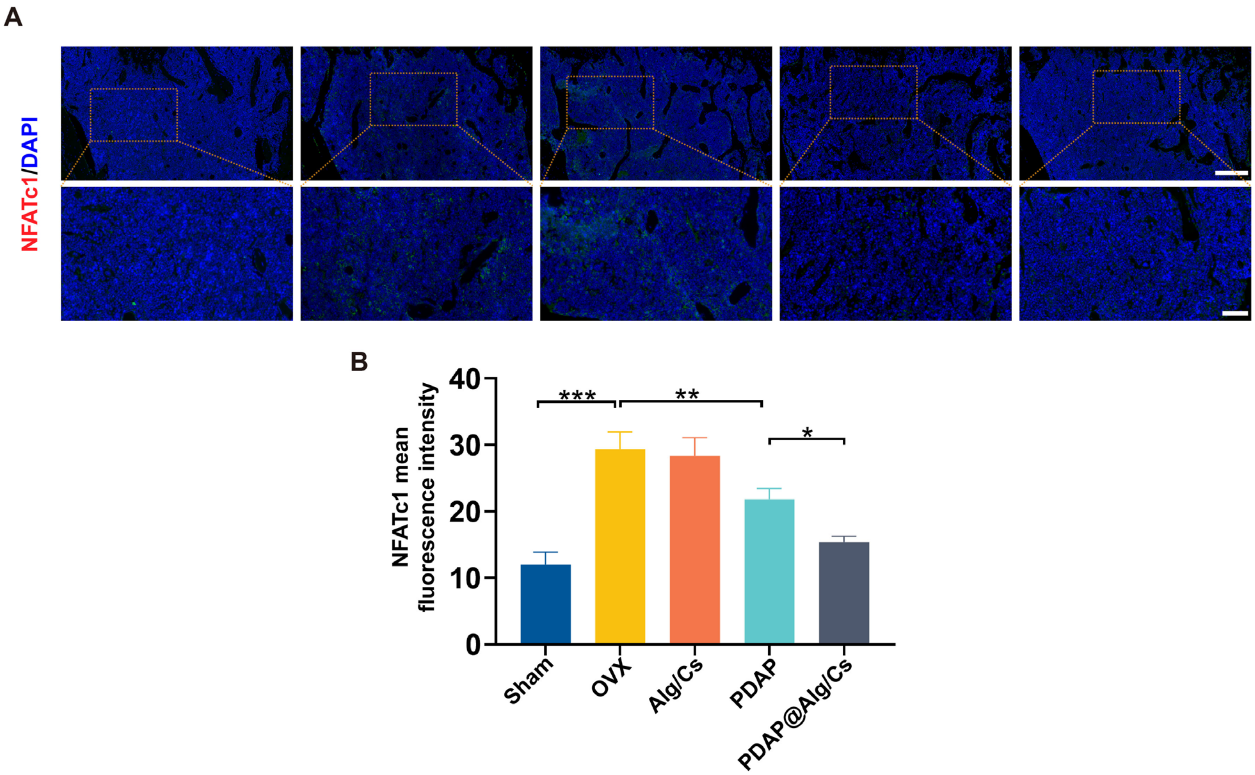

Fig. S12. (A) Immunofluorescence staining of NFATc1 in the distal femur. Scale bars, 200 μm and 50 μm for top and bottom images, respectively. (B) Quantification of the NFATc1 mean fluorescence intensity in different groups (n=6).

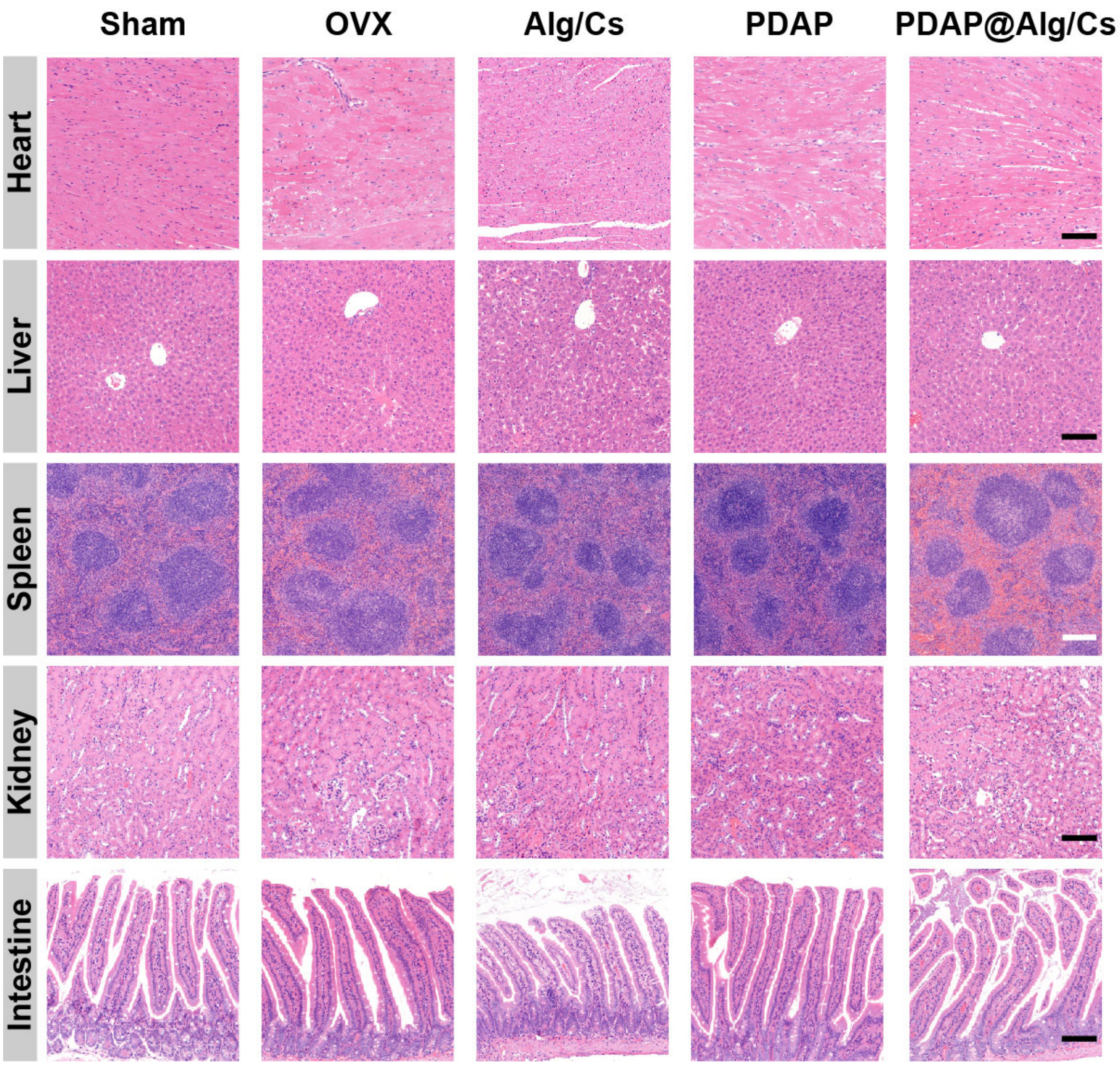

Fig. S13. Representative H&E images of heart, liver, spleen, kidney, and intestine sections in different groups. Scale bars, 100 μm and 200 μm for black and white bars, respectively.

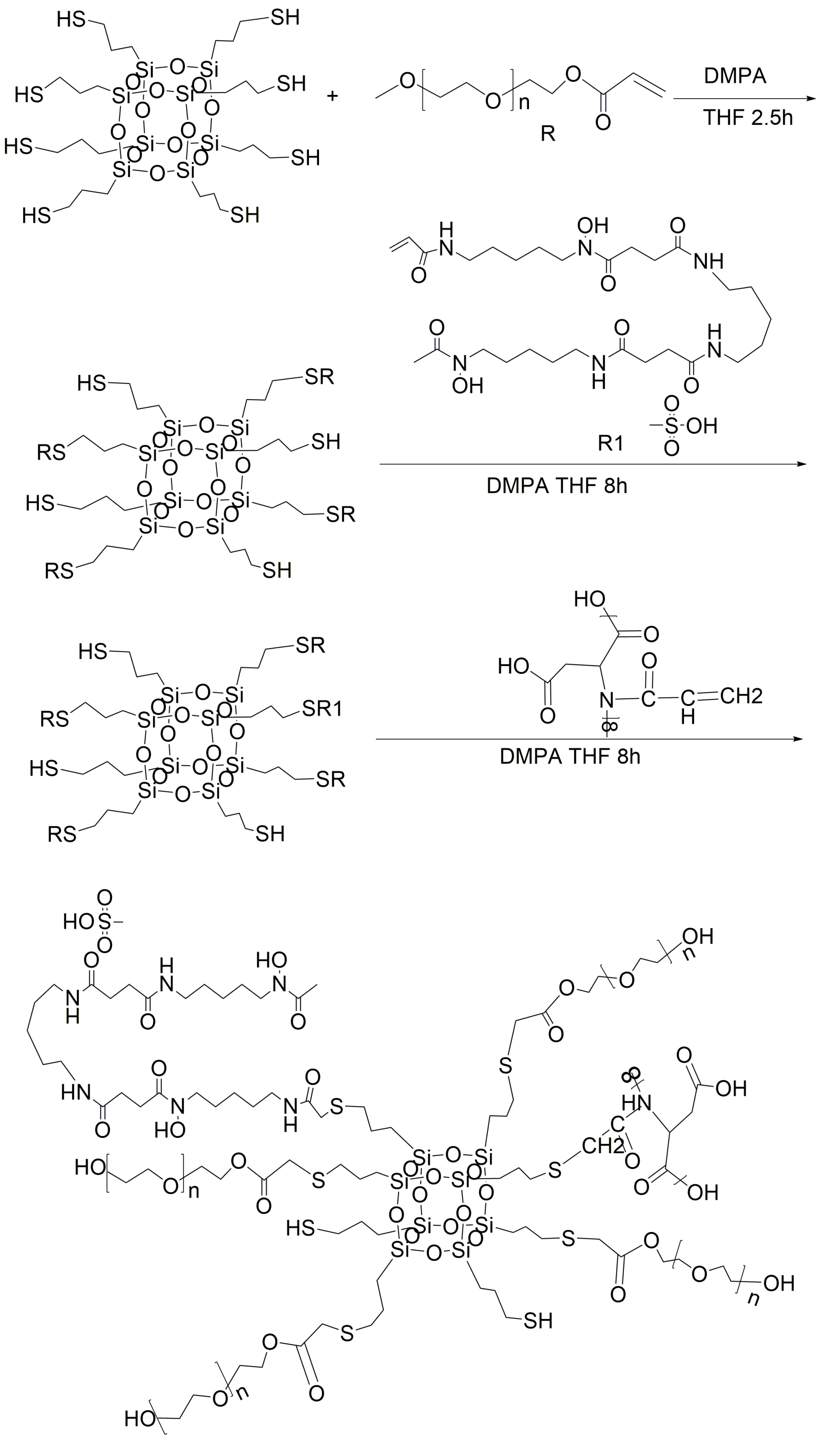

Fig. S14. Schematic synthesis of PDAP NPs.

Table S1. Sequences of primers used for the real-time quantitative PCR analysis.

Primers	Forward	Reverse
Mouse *Trap*	5'-TACCTGTGTGGACATGACC-3'	5'- CAGATCCATAGTGAAACCGC-3'
Mouse *Ctsk*	5'-GAAGAAGACTCACCAGAAGCAG-3'	5'-TCCAGGTTATGGGCAGAGATT-3'
Mouse *Nfatc1*	5'-GACCCGGAGTTCGACTTCG-3'	5'-TGACACTAGGGGACACATAACTG-3'
Mouse *Ho-1*	5'-AAGCCGAGAATGCTGAGTTCA-3'	5'-GCCGTGTAGATATGGTACAAGGA-3'
Mouse *β-actin*	5'-GGCTGTATTCCCCTCCATCG-3'	5'- CCAGTTGGTAACAATGCCATGT-3'
Human *VEGF*	5'-AGGGCAGAATCATCACGAAGT-3'	5'-AGGGTCTCGATTGGATGGCA-3'
Human *HIF-1α*	5'-CCATGTGACCATGAGGAAAT-3'	5'-CGGCTAGTTAGGGTACACTT-3'
Human *β-ACTIN*	5'-CATGTACGTTGCTATCCAGGC-3'	5'-CTCCTTAATGTCACGCACGAT-3'
